# Neuronal GPCR NPR-8 regulates *C. elegans* defense against pathogen infection

**DOI:** 10.1126/sciadv.aaw4717

**Published:** 2019-11-20

**Authors:** Durai Sellegounder, Yiyong Liu, Phillip Wibisono, Chia-Hui Chen, David Leap, Jingru Sun

**Affiliations:** 1 Department of Biomedical Sciences, Elson S. Floyd College of Medicine, Washington State University, Spokane, WA, USA.; 2 Genomics Core, Washington State University, Spokane, WA, USA.

## Abstract

Increasing evidence indicates that infection-triggered host defenses are regulated by the nervous system. However, the precise mechanisms of this regulation are not well understood. Here, we demonstrate that neuronal G protein-coupled receptor NPR-8 negatively regulates *Caenorhabditis elegans* defense against pathogen infection by suppressing cuticular collagen expression. NPR-8 controls the dynamics of cuticle structure in response to infection, likely through its regulation of cuticular collagen genes which, in turn, affects the nematode’s defense. We further show that the defense activity of NPR-8 is confined to amphid sensory neurons AWB, ASJ, and AWC. It is generally believed that physical barrier defenses are not a response to infections but are part of the body’s basic innate defense against pathogens. Our results challenge this view by showing not only that *C. elegans* cuticle structure dynamically changes in response to infection but also that the cuticle barrier defense is regulated by the nervous system.

## Teaser

The nervous system regulates *C. elegans* defense against pathogen infection by controlling the dynamics of cuticle structure.

## INTRODUCTION

Host defense against pathogen infection involves the use of physical barriers as well as behavior and physiological responses to resist, reduce, and kill invading pathogens (*1*). The genetically tractable model host *Caenorhabditis elegans* offers a valuable tool to dissect the mechanisms of host defense. *C. elegans* is a free-living nematode found in soil and decaying organic matter, where it feeds on bacteria and is inevitably attacked by pathogenic microbes. The nematode’s cuticle and gut epithelial cells are physical barriers that form the first line of defense to prevent pathogens from entering the body (*2*). Certain pathogens can also induce behavioral changes in *C. elegans*, including direct avoidance behaviors, reduced oral uptake of pathogens, and a learning response that enhances avoidance upon exposure to the same pathogens for a second time (*3*, *4*). Despite lacking adaptive immunity, *C. elegans* has an immune system that resembles the human innate immune system in several key respects and can trigger evolutionarily conserved immune signaling pathways (*5*). Activation of these pathways induces the expression of defense genes such as those encoding lectins, lysozymes, lipases, and antimicrobial peptides, which then act directly or indirectly to combat invading microbes.

Infection-triggered host defense responses must be tightly controlled because insufficient responses exacerbate infection, whereas excessive responses lead to prolonged inflammation, tissue damage, or even death (*6*). Recent studies indicate that the nervous system regulates defense responses including behavioral changes and immune responses to help maintain healthy homeostasis (*7*). However, the mechanisms of this regulation are not well understood. Nevertheless, research on neural defense regulation using the *C. elegans* model is at the forefront of this field mainly because of the nematode’s simple and well-defined nervous system and because of its use as a model host for bacterial pathogenesis research. Studies in *C. elegans* have led to the identification of specific G protein–coupled receptors (GPCRs), neurotransmitters, neuropeptides, neurons, and nonneural cells in the regulation of host defense. For example, *C. elegans* uses the olfactory sensory neurons AWA, AWB, and AWC to sense different odorants and other cues from bacterial food and pathogens and initiate attractive or repulsive responses toward these bacteria (*3*, *4*). Several neural circuits that regulate innate immune responses have also been discovered. We and the Aballay group (*8*–*10*) have revealed that octopamine originating in RIC neurons acts as a ligand of the GPCR OCTR-1 that functions in the sensory ASH neurons to suppress innate immune responses in pharyngeal and intestinal tissues. Anderson *et al.* (*11*) demonstrated that upon infection of *C. elegans* with the bacterial pathogen *Microbacterium nematophilum*, ADF neurons release serotonin, which then acts via its receptors SER-1 and SER-7, to suppress the innate immune response in the rectal epithelium. Chemical inhibition of dopamine in CEP neurons enhances innate immunity through the D1-like dopamine receptor DOP-4 in ASG neurons (*12*). Although many details are still lacking in these neural immunoregulatory circuits, their discovery has greatly facilitated our understanding of neural regulation of defense responses.

GPCRs are the key players in neural defense regulation. GPCRs are a large family of transmembrane proteins that sense many extracellular signals and transduce them into cellular physiological responses. The neuropeptide Y (NPY) receptors, a subfamily of GPCRs produced in the nervous system, have notable roles in regulating behaviors and the immune system (*13*). In higher vertebrates, NPY receptors display bimodular function by suppressing the innate immune system and by activating antigen-presenting cells (*14*). *C. elegans* contains 41 NPY/RFamide-like receptors, among which 12 have been previously studied (*15*), and the others have yet to be explored. One such putative receptor is C56G3.1, which corresponds to the *npr-8* gene (Wormbase). Styer *et al.* (*16*) screened 40 *C. elegans* strains carrying mutations in GPCRs by examining their susceptibility to *Pseudomonas aeruginosa* infection and found that loss-of-function mutations in three GPCR genes conferred enhanced survival to the nematode; *npr-8* is one of those GPCR genes. However, the functionality of NPR-8 and its mode of signaling in *C. elegans* defense are unknown. In the current study, we characterize the roles of NPR-8 in *C. elegans* defense against pathogen infection. We have found that NPR-8 negatively regulates nematode defense by suppressing cuticular collagen expression. NPR-8 controls the dynamics of cuticle structure in response to infection, which is likely achieved through its regulation of cuticular collagen genes and affects the nematode’s defense. NPR-8’s activity in defense is confined to the amphid sensory neurons AWB, ASJ, and AWC, but it is not involved in bacteria sensing in these neurons. These results suggest that the cuticle structure dynamically changes in response to pathogen infection and that the cuticle barrier response is regulated by the nervous system. Our results challenge a general view that physical barrier defenses are not a response to infections but are part of the body’s basic defense that is continuously working to protect against pathogens (*1*).

## RESULTS

### Functional loss of NPR-8 enhances *C. elegans* survival against pathogen infection

**Fig. 1:**
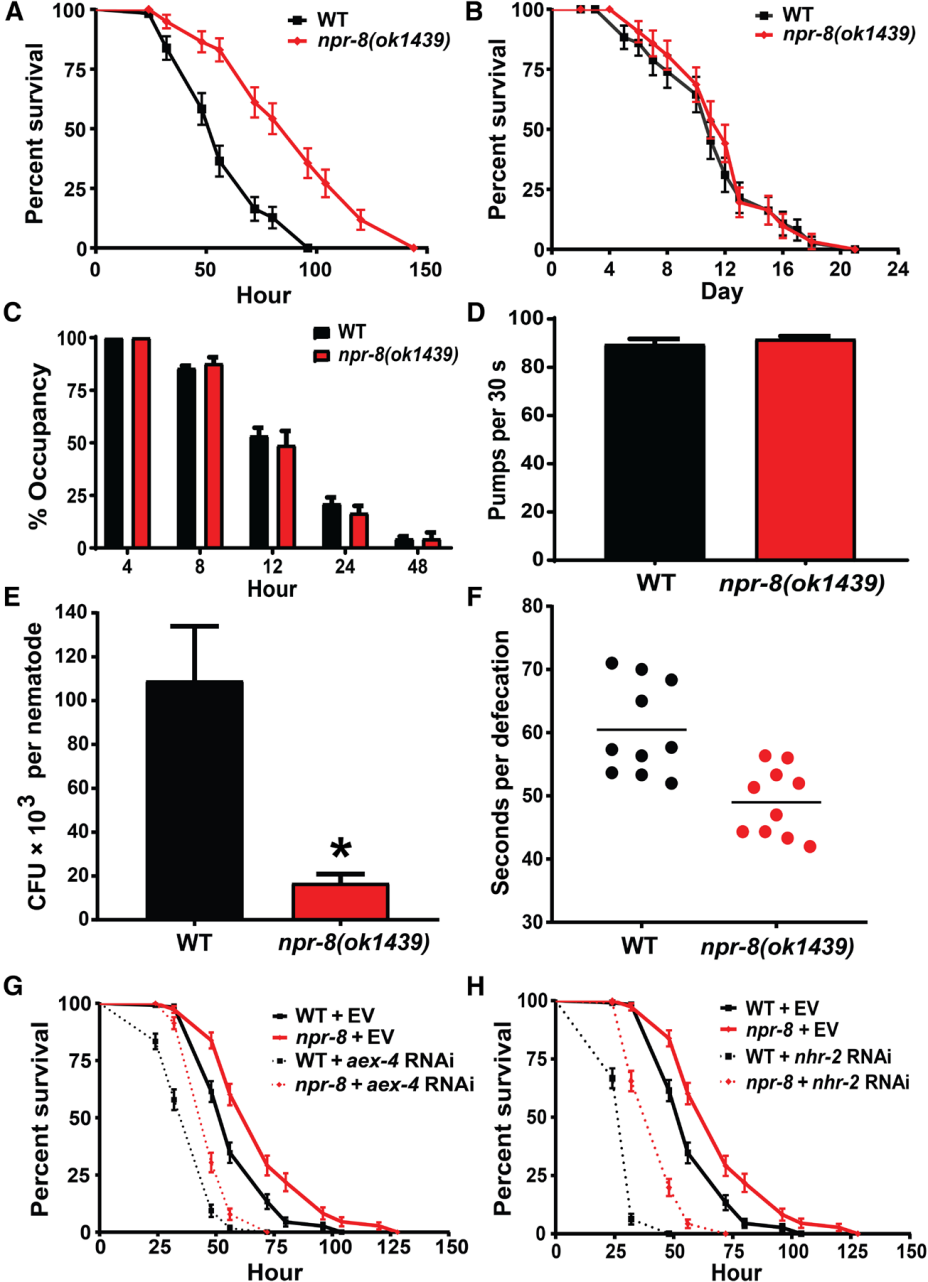
Functional loss of NPR-8 enhances *C. elegans* survival against pathogen infection and increases pathogen clearance from the intestine. (**A**) Wild-type (WT) and *npr-8(ok1439)* animals were exposed to *P. aeruginosa* and scored for survival over time. The graphs are the combined results of three independent experiments. Each experiment included *n* = 60 adult animals per strain. Error bars represent the SEM. *P* value represents the significance level of the mutants relative to the WT, *P* < 0.0001. (**B**) WT and *npr-8(ok1439)* animals were exposed to heat-killed *P. aeruginosa* and scored for survival over time. The graphs are the combined results of two independent experiments. Each experiment included *n* = 60 adult animals per strain. Error bars represent SEM. *P* value represents the significance level of the mutants relative to the WT, *P* = 0.50. (**C**) WT and *npr-8(ok1439)* animals were subjected to lawn occupancy assays in which animals were placed on a small spot of *P. aeruginosa* in a 3.5-cm plate and monitored over time for their presence on the lawn. The graphs are the combined results of three independent experiments. Each experiment included *n* = 60 adult animals per strain. Error bars represent SEM. *P* values represent the significance level of the mutants relative to the WT, *P* > 0.05, for all five time points. (**D**) WT and *npr-8(ok1439)* animals were exposed to *P. aeruginosa* in full lawn plates for 24 hours. Pharyngeal pumping rates of animals were counted as the number of pumps per 30 s. Counting was conducted in triplicate and averaged to give a pumping rate. The graphs are the combined results of three independent experiments. Each experiment included *n* = 60 adult animals per strain. Error bars represent SEM. *P* value represents the significance level of the mutants relative to WT, *P* = 0.3934. (**E**) WT and *npr-8(ok1439)* animals were exposed to GFP-expressing *P. aeruginosa* in full lawn plates for 24 hours, and the CFU of live bacterial cells recovered from the intestine were quantified. The graph represents the combined results of three independent experiments. *n* = 10 animals per strain were used for each experiment. Error bars represent SEM. Asterisk (*) denotes a significant difference (*P* < 0.05) between WT and *npr-8(ok1439)* animals. (**F**) WT and *npr-8(ok1439)* animals were exposed to *P. aeruginosa* in full lawn plates for 24 hours. Defecation rates of animals were measured as the average time of 10 intervals between two defecation cycles. *n* = 10 animals per strain were used for the measurements. *P* value represents the significance level of the mutants relative to WT, *P* = 0.0003. (**G**) WT and *npr-8(ok1439)* animals grown on double-stranded RNA (dsRNA) for empty vector (EV) control or dsRNA for *aex-4* were exposed to *P. aeruginosa* and scored for survival over time. The graphs are the combined results of two independent experiments. Each experiment included *n* = 60 adult animals per strain. Error bars represent the SEM. *P* value represents the significance level: WT + EV versus *npr-8(ok1439)* + EV, *P* < 0.0001; WT + *aex-4* RNAi versus *npr-8(ok1439)* + *aex-4* RNAi, *P* < 0.0001. (**H**) WT and *npr-8(ok1439)* animals grown on dsRNA for EV control or dsRNA for *nhr-2* were exposed to *P. aeruginosa* and scored for survival over time. The graphs are the combined results of two independent experiments. Each experiment included *n* = 60 adult animals per strain. Error bars represent the SEM. *P* value represents the significance level: WT + EV versus *npr-8(ok1439)* + EV, *P* < 0.0001; WT + *nhr-2* RNAi versus *npr-8(ok1439)* + *nhr-2* RNAi, *P* < 0.0001.

To understand the roles of NPR-8 in *C. elegans* defense against pathogen infection, we examined the survival of *C. elegans* lacking NPR-8 [*npr-8(ok1439)* null animals] following exposure to the human opportunistic pathogen *P. aeruginosa* strain PA14. Compared to wild-type N2 animals, *npr-8(ok1439)* animals exhibited enhanced survival against *P. aeruginosa* (Fig. 1A). We observed no difference in survival between *npr-8(ok1439)* and wild-type animals that were fed heat-killed *P. aeruginosa* (Fig. 1B) or heat-killed or live *Escherichia coli* strain OP50 (fig. S1, A and B). These results indicate that the *npr-8* mutation affects *C. elegans* defense against living pathogenic bacteria without altering the basic life span of the nematode. The survival of *npr-8(ok1439)* animals following pathogen infection was further studied with two additional paradigmatic human pathogens: the Gram-negative bacterium *Salmonella enterica* strain SL1344 and the Gram-positive bacterium *Staphylococcus aureus* strain MSSA476. Loss of NPR-8 enhances the nematode’s survival against both bacterial pathogens (fig. S1, C and D), demonstrating that NPR-8 suppresses *C. elegans* defense against multiple pathogens.

Certain mutations in *C. elegans* can cause sterility, resulting in increased resistance to pathogen infection (*17*). During our assays, we did not observe any sterility phenotypes displayed by *npr-8(ok1439)* animals. The mutants produced viable progeny, and their brood size was similar to that of wild-type animals at both the growth temperature 20°C and the survival assay temperature 25°C. To determine whether the enhanced survival phenotype of *npr-8* mutants is allele specific, we performed survival assays using another *npr-8* null strain [*npr-8(ok1446)* animals] that contains a deletion mutation in the *npr-8* gene at a location different from that in *npr-8(ok1439)* animals (*18*). Results showed that, like *npr-8(ok1439)* animals, *npr-8(ok1446)* animals exhibited enhanced survival against *P. aeruginosa* compared to wild-type animals (fig. S1E). These results indicate that the enhanced survival phenotype caused by *npr-8* mutations is not allele specific.

To investigate the possible involvement of sterility in the enhanced survival of *npr-8(ok1439)* animals, we induced sterility in both wild-type and *npr-8*(*ok1439*) animals by using RNA interference (RNAi) to inactivate *cdc-25.1*, an important gene for germline proliferation (*19*). RNAi was conducted by feeding the animals *E. coli* strain HT115 (DE3) with a vector expressing double-stranded RNA (dsRNA) that is homologous to a target gene or with an empty vector as a negative control (*20*). Survival assays against *P. aeruginosa* showed that RNAi of *cdc-25.1* increased the life span of both wild-type and *npr-8(ok1439)* animals for about fourfold but did not change the enhanced survival phenotype of *npr-8(ok1439)* animals relative to wild-type animals (fig. S1F). The difference in survival between the two strains was clear until 300 hours, after which they died at a similar rate, possibly due to aging (fig. S1F). These results suggest that sterility does not play a causal role in the enhanced survival phenotype of *npr-8(ok1439)* animals.

The enhanced survival of *npr-8(ok1439)* animals could be due to altered pathogen avoidance behavior, a decrease in pathogen intake or accumulation, and/or an increase in innate immunity. These possible causes were further investigated, and the results are described below.

### Pathogen avoidance behavior does not contribute to the improved survival of *npr-8(ok1439)* animals

*C. elegans* exhibits avoidance behavior in the presence of certain types of pathogenic bacteria including *P. aeruginosa*, and this change in behavior is considered part of its defense mechanisms (*3*). To test whether pathogen avoidance plays a role in the enhanced survival of *npr-8(ok1439)* animals, we measured their survival using full-lawn assays in which agar plates were completely covered by pathogenic bacteria so that pathogen avoidance was eliminated. Like our previous observations, *npr-8(ok1439)* animals exposed to *P. aeruginosa* died at a slower rate than the wild-type controls (fig. S1G), suggesting that lawn avoidance is not the reason for the improved survival. This notion is also supported by the results of our previous survival assays showing that *npr-8(ok1439)* animals exhibit enhanced survival following exposure to *S. enterica* (fig. S1C), a pathogen that does not elicit an avoidance behavior (*21*). We next conducted lawn occupancy behavioral assays to compare the magnitude of pathogen avoidance between *npr-8(ok1439)* animals and wild-type animals. In this assay, a small lawn of *P. aeruginosa* was cultured in the center of an agar plate, and the number of animals that stayed on and off the lawn was counted at five time points over a period of 48 hours. Compared to wild-type animals, the mutant animals displayed no significant differences in the magnitudes of pathogen avoidance (Fig. 1C). Overall, these results indicate that pathogen avoidance does not contribute to the improved survival of the *npr-8* mutant animals.

### Functional loss of NPR-8 increases clearance of *P. aeruginosa* from the intestine

The improved survival of *npr-8(ok1439)* animals could be due to a reduction in pathogen intake and/or pathogen accumulation. *C. elegans* feeds on bacterial food via rhythmic contractions (pumping) of its pharynx. To determine whether there is any difference in pathogen intake between *npr-8(ok1439)* and wild-type animals, we measured the pharyngeal pumping rates of the animals on bacterial lawns. When feeding on *P. aeruginosa*, the mutant animals showed pumping rates similar to wild-type animals (Fig. 1D), indicating similar levels of pathogen intake. Because certain mutations in *C. elegans* can cause reduced pathogen accumulation in the intestine, resulting in enhanced resistance to pathogen infection (*22*), we then examined whether the *npr-8* mutation affects bacterial accumulation. We used green fluorescent protein (GFP)–tagged *P. aeruginosa* PA14 to visualize the fluorescence intensity of accumulated bacteria in the nematode’s intestine. Our results showed substantial reductions in GFP intensity in *npr-8(ok1439)* animals compared to wild-type animals (fig. S1H). Intestinal bacterial loads were also quantified by counting the colony-forming units (CFUs) of live bacterial cells recovered from the intestines of wild-type and mutant animals (*8*). The mutant animals showed significantly fewer CFUs of *P. aeruginosa* than wild-type animals (Fig. 1E), indicating that the *npr-8* mutation causes a reduction in bacterial accumulation. This reduction, following wild type–level pathogen intake, suggests an increased ability of the mutants to clear *P. aeruginosa* infection from the intestine. We next tested whether bacterial evacuation contributes to the increased pathogen clearance ability in mutant animals. The rate of bacterial evacuation can be estimated by the defecation rate, defined as the time interval between expulsions of gut contents. We found that the defecation cycle interval of *npr-8(ok1439)* animals on *P. aeruginosa* was significantly shorter than that of wild-type animals (Fig. 1F), meaning that the mutants defecate more often. These results indicate that *npr-8* mutant animals could maintain less bacterial load through, or partially through, faster defecation.

To determine whether faster defecation contributes to the enhanced survival of *npr-8(ok1439)* animals, we inactivated the *C. elegans* defecation motor program (DMP) and tested whether the inactivation rescues the enhanced survival phenotype of *npr-8* mutant animals. To this end, two genes (*aex-4* and *nhx-2*) that regulate the DMP were selected for inactivation because inhibition of these genes is known to cause defective DMP (*23*). These genes were individually knocked down by RNAi in wild-type and *npr-8(ok1439)* animals, followed by survival assays against *P. aeruginosa*–mediated killing. Results showed that inactivation of either of these two genes substantially reduced the survival of both wild-type and *npr-8(ok1439)* animals, but in both cases, the *npr-8* mutants were significantly more resistant to infection than wild-type animals (Fig. 1, G and H). These results indicate that although defecation has a great influence on survival in general, it is not the cause of the enhanced survival of *npr-8(ok1439)* animals. Therefore, increased defecation rate and enhanced survival against infection are likely two independent phenotypes caused by the *npr-8* null mutation.

The increased defecation rate of *npr-8(ok1439)* animals suggests that NPR-8 may function in defecation-regulating neurons to suppress defecation activity. Two GABAergic neurons [neurons that generate neurotransmitter γ-aminobutyric acid (GABA)], AVL and DVB, are involved in regulating the *C. elegans* DMP (*23*). To determine whether NPR-8 functions in these neurons to regulate defecation and survival against infection, we rescued *npr-8* expression in *npr-8(ok1439)* animals using the *aex-2* promoter that drives gene expression in the two GABAergic neurons and enteric muscles (*23*). The rescue strain JRS25, along with wild-type and *npr-8(ok1439)* animals, was then tested for defecation rate and survival against *P. aeruginosa* infection. Results showed that rescuing *npr-8* expression in the GABAergic neurons significantly suppressed the increased defecation rate of *npr-8(ok1439)* animals (fig. S2A) but did not suppress their enhanced survival (fig. S2B). These results indicate that NPR-8 likely functions in AVL and DVB neurons to control defecation, and that function is independent of its activity in defense against infection, which agrees with the above-described DMP inactivation experiments.

### NPR-8 does not play a role in conserved innate immune pathways

The enhanced survival of *npr-8(ok1439)* animals could be caused by increased innate immunity. Upon pathogen infection, *C. elegans* can mount protective responses by triggering evolutionarily conserved innate immune signaling pathways, such as the PMK-1/p38 mitogen-activated protein kinase (MAPK) pathways, the DBL-1 pathway [homologous to the mammalian transforming growth factor–β (TGF-β) cascade], and the unfolded protein response (UPR) (*24*–*26*). To investigate whether NPR-8 is involved in immune regulation, we examined how the *npr-8* mutation affects gene expression in these signaling pathways in response to pathogen infection. To this end, we performed quantitative reverse transcription polymerase chain reaction (qRT-PCR) to measure transcript expression levels of 5 to 10 representative genes in each of the abovementioned three pathways (*24*–*26*). The expression of most of the genes tested was not significantly changed in *npr-8(ok1439)* animals relative to wild-type animals exposed to *P. aeruginosa* (fig. S3). More specifically, 6 genes in the PMK-1 pathway were tested and only 1 (*F52F10.4*) was significantly up-regulated (fig. S3A), none of the 10 genes we tested in the TGF-β pathway showed significant changes in expression (fig. S3B), and one of the 5 genes tested in the UPR pathway was significantly down-regulated (fig. S3C). Because most of the genes tested in these conserved immune pathways were not affected by the *npr-8* mutation, it is unlikely that NPR-8 is a regulator of any of these pathways. However, the two individual genes that were affected could be involved in other unknown biological processes that are modulated by NPR-8.

We also performed RNA sequencing (RNA-seq) to profile gene expression in *npr-8(ok1439)* animals relative to wild-type animals exposed to *P. aeruginosa*. The RNA-seq analyses are described in detail in the next section. To determine whether NPR-8 plays a role in the abovementioned three immune pathways, we examined the 10 most highly regulated target genes in each of the three pathways reported by three original studies [the PMK-1 pathway by Troemel *et al.* (*24*), the TGF-β pathway by Mochii *et al.* (*25*), and the UPR pathway by Shen *et al.* (*26*)]. Our RNA-seq data showed that these genes had little to no change in expression in *npr-8(ok1439)* animals relative to wild-type animals exposed to *P. aeruginosa* (table S1); for genes that did show changes in expression, none of the changes exceed twofold, an arbitrary cutoff value above which was considered as significant enrichment in our RNA-seq data analyses (table S1). These results are consistent with the above-described qRT-PCR results, confirming that NPR-8 is unlikely a regulator of the immune pathways.

### NPR-8 regulates *C. elegans* defense against *P. aeruginosa* by suppressing cuticular collagen genes

To gain insights on how NPR-8 regulates *C. elegans* defense at the molecular level, we used RNA-seq to profile gene expression in *npr-8(ok1439)* animals relative to wild-type animals with or without *P. aeruginosa* infection. To this end, we collected five replicates of four groups of RNA samples [*npr-8(ok1439)* and wild-type animals with or without exposure to *P. aeruginosa* for 24 hours]. These RNA samples were then submitted to the Washington State University (WSU) Genomics Core for RNA-seq analyses. The resulting sequence data have been deposited in the National Center for Biotechnology Information’s (NCBI) Sequence Read Archive (SRA) database through the Gene Expression Omnibus (GEO); processed gene quantification files and differential expression files have been deposited in GEO; and all of these data can be accessed through GEO with the accession number GSE122544.

To assess the effectiveness of RNA-seq in our study, we compared the expression profile of *P. aeruginosa*–infected wild-type animals with that of uninfected controls. In total, 14,126 genes were identified and quantified with a false discovery rate (FDR) of 5%. Among these genes, 3908 were up-regulated at least twofold and 1810 were down-regulated at least twofold in infected wild-type animals relative to uninfected controls (GEO accession number GSE122544). Gene ontology (GO) analysis of the 3839 up-regulated genes identified 21 significantly enriched biological processes, 20 of which involve responses to infection or other external stimuli (table S2A). This is consistent with the use of the *P. aeruginosa–C. elegans* model for bacterial pathogenesis research (*5*).

To examine how the lack of NPR-8 in *C. elegans* affects gene expression under normal conditions (i.e., without infection), we compared the expression profile of *npr-8(ok1439)* animals with that of wild-type animals. We found that 135 genes were up-regulated at least twofold in the mutant animals, with no genes down-regulated more than twofold (GEO accession number GSE122544). Ontology analysis of the up-regulated genes revealed two significantly enriched molecular functions, both of which involve cuticle structure activity (table S2B). Thirty-five genes identified are related to this activity, 34 of which encode cuticular collagens (table S2C). The nematode cuticle is an exoskeleton composed predominantly of cross-linked collagens and serves as a permeability barrier and first line of defense against environmental assault, including pathogenic attack (*27*). It is synthesized five times, once in the embryo and subsequently at the end of each larval stage before molting. Because these collagen genes were identified in adult animals, changes in juvenile molting are not the cause of their differential expression. These results indicate that NPR-8 negatively regulates the adult cuticle structure under normal conditions. The lack of NPR-8–dependent suppression of cuticular collagen genes in *npr-8(ok1439)* animals might contribute to their improved survival against pathogen infection.

Next, we asked how NPR-8 regulates gene expression in *C. elegans* in response to pathogen infection. We examined the gene expression profile of *npr-8(ok1439)* animals relative to wild-type animals exposed to *P. aeruginosa* and found that 210 genes were up-regulated at least twofold and 13 genes were down-regulated at least twofold (GEO accession number GSE122544). GO analysis of the up-regulated genes identified one significantly enriched molecular function, cuticle structure activity (Table 1A). This enrichment resulted from the induction of 12 cuticular collagen genes in the mutant animals (Table 1B). Most of these genes were slightly induced by the *npr-8* null mutation under uninfected conditions and were further up-regulated upon *P. aeruginosa* exposure (Table 1B). These results indicate that regulation of cuticle structure by NPR-8 could play a role in the nematode’s defense against *P. aeruginosa*.

**Table 1:** Cuticular collagen genes were up-regulated in *npr-8(ok1439)* animals relative to wild-type animals exposed to *P. aeruginosa*.

**A. Enrichment of molecular functions revealed by GO analysis of up-regulated genes in *npr-8(ok1439)* animals relative to wild-type animals exposed** **to *P. aeruginosa.***						
**GO term**	**Description**		P*	**FDR *q*^†^**	**Enrichment (*N*, *B*, *n*, *b*)^‡^**	
GO:0042302	Structural constituent of cuticle		3.08 × 10^−8^	6.87 × 10^−5^	7.96 (10,359, 121, 129, 12)	
**B. Enrichment of cuticular collagen genes in *npr-8(ok1439)* animals relative to wild-type animals with or without exposure to *P. aeruginosa*.**						
**Enriched collagen** **genes**	**Infected with *P. aeruginosa***			**Uninfected**		
	***npr-8(ok1439)* animals relative to wild-type animals**			***npr-8(ok1439)* animals relative to wild-type animals**		
	**Fold change**	**Adjusted *P*^§^**	**Significantly** **different?**	**Fold change**	**Adjusted *P*^§^**	**Significantly** **different?**
*col-93*	4.6	3.75 × 10^−82^	Yes	1.3	2.82 × 10^−2^	Yes
*col-80*	3.5	2.03 × 10^−38^	Yes	1.3	2.17 × 10^−2^	Yes
*col-179*	2.5	1.31 × 10^−22^	Yes	1.2	1.09 × 10^−1^	No
*col-98*	2.4	6.85 × 10^−22^	Yes	1.3	4.35 × 10^−2^	Yes
*col-160*	2.4	9.91 × 10^−33^	Yes	1.4	1.37 × 10^−3^	Yes
*col-124*	2.4	2.97 × 10^−14^	Yes	1.2	9.33 × 10^−2^	No
*col-103*	2.2	1.35 × 10^−17^	Yes	1.3	3.74 × 10^−2^	Yes
*col-101*	2.1	8.11 × 10^−14^	Yes	1.2	1.78 × 10^−4^	Yes
*col-146*	2.0	9.72 × 10^−8^	Yes	3.3	9.24 × 10^−23^	Yes
*col-159*	2.0	1.20 × 10^−6^	Yes	1.8	3.05 × 10^−6^	Yes
*col-20*	2.0	2.13 × 10^−16^	Yes	1.3	4.75 × 10^−2^	Yes
*Y69H2.14*	2.0	1.13 × 10^−14^	Yes	1.3	6.05 × 10^−2^	No

*P value is the enrichment *P* value computed according to the mHG model (Eden *et al.* 2007 PLoS Comp Bio 3 (3):e39).

†FDR *q* value is the correction of the above *P* value for multiple testing using the Benjamini and Hochberg method (Benjamini and Hochberg 1995 J R Statist Soc B 57 (1):289–300).

‡Enrichment (*N*, *B*, *n*, *b*) is defined as follows: *N*, total number of genes; *B*, total number of genes associated with a specific GO term; *n*, number of genes in the target set; *b*, number of genes in the intersection; Enrichment = (*b*/*n*)/(*B*/*N*).

§Adjusted *P* value is the correction of the *P* value for multiple testing using the Benjamini and Hochberg method (Benjamini and Hochberg 1995 J R Statist Soc B 57 (1):289–300).

**Fig. 2:**
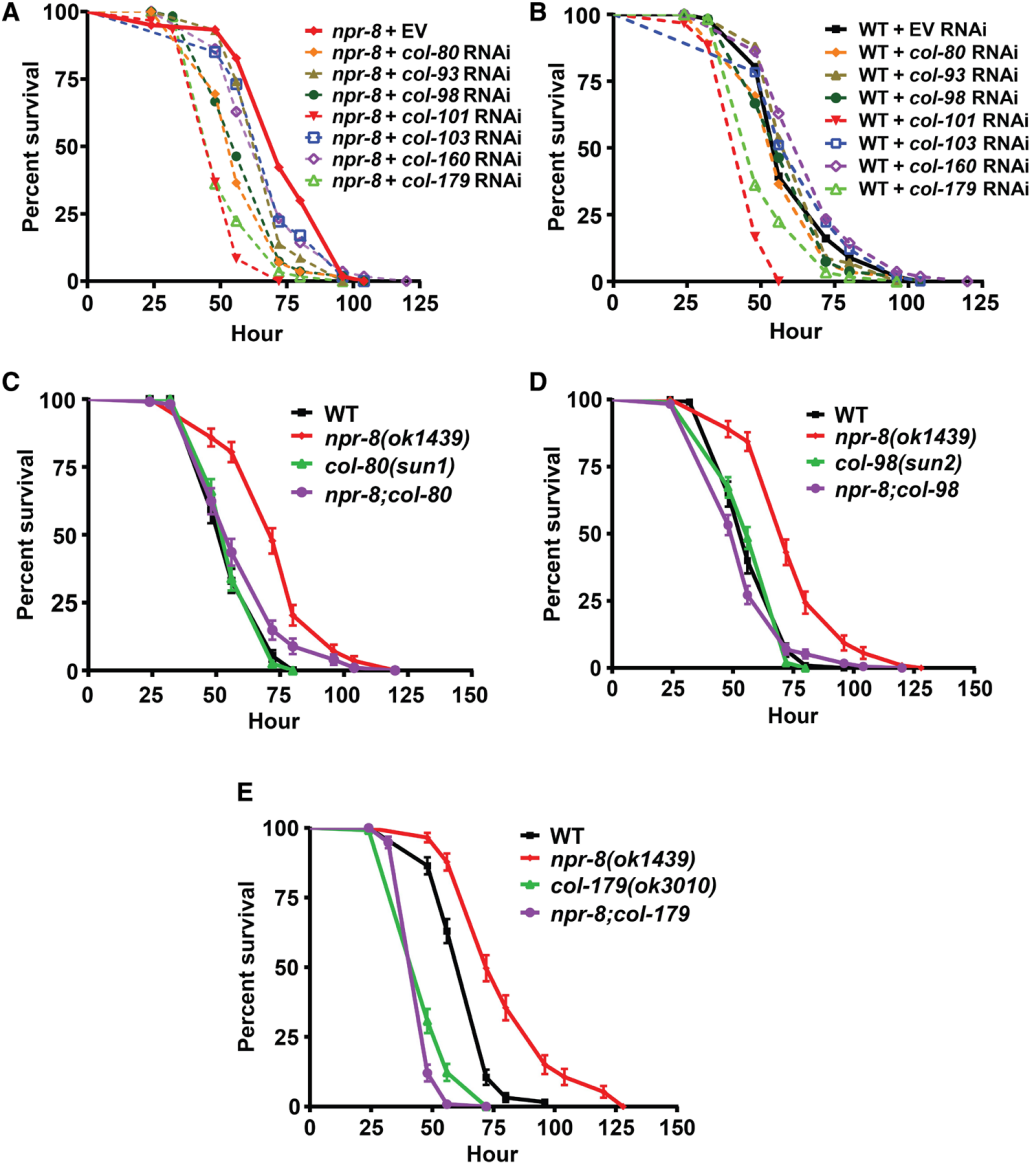
NPR-8-regulated collagen genes are required for *C. elegans* defense against *P. aeruginosa* infection. (**A**) *npr-8(ok1439)* and (**B**) WT animals grown on dsRNA for collagen genes or EV control were exposed to ***P. aeruginosa*** and scored for survival over time. The graphs are the combined results of three independent experiments. Each experiment included *n* = 60 adult animals per strain. *P* values in (A) are relative to *npr-8* + EV*: npr-8* + *col-80* RNAi, *P* < 0.0001; *npr-8 + col-93* RNAi, *P* = 0.0030; *npr-8 + col-98* RNAi, *P* < 0.0001; *npr-8 + col-101* RNAi, *P* < 0.0001; *npr-8 + col-103* RNAi, *P* = 0.0601; *npr-8 + col-160* RNAi, *P* = 0.0333; *npr-8 + col-179* RNAi, *P* < 0.0001. *P* values in (B) are relative to WT + EV: WT + *col-80* RNAi, *P* = 0.2555; WT + *col-93* RNAi, *P* = 0.5575; WT + *col-98* RNAi, *P* = 0.5250; WT + *col-101* RNAi, *P* < 0.0001; WT + *col-103* RNAi, *P* = 0.6950; WT + *col-160* RNAi, *P* = 0.0494; WT + *col-179* RNAi, *P* = 0.0004. (**C**) WT, *npr-8(ok1439)*, *col-8(sun1)*, and *npr-8;col-80* animals were exposed to *P. aeruginosa* and scored for survival over time. *P* values are as follows: WT versus *npr-8(ok1439)*, *P* < 0.0001; WT versus *col-80(sun1)*, *P* = 0.8761; *npr-8(ok139)* versus *npr-8;col-80*, *P* < 0.0001. (**D**) WT, *npr-8(ok1439)*, *col-98(sun2)*, and *npr-8;col-98* animals were exposed to *P. aeruginosa* and scored for survival over time. *P* values are as follows: WT versus *npr-8(ok1439)*, *P* < 0.0001; WT versus *col-98(sun2)*, *P* = 0.7044; *npr-8(ok139)* versus *npr-8;col-98*, *P* < 0.0001. (**E**) WT, *npr-8(ok1439)*, *col-179(ok3010)*, and *npr-8;col-179* animals were exposed to *P. aeruginosa* and scored for survival over time. *P* values are as follows: WT versus *npr-8(ok1439)*, *P* < 0.0001; WT versus *col-179(ok3010)*, *P* < 0.0001; *npr-8(ok139)* versus *npr-8;col-179*, *P* < 0.0001.

To examine whether the NPR-8–regulated cuticular collagen genes contribute to the improved survival of the mutant animals, we inactivated these genes by RNAi in wild-type and *npr-8(ok1439)* animals and assayed their survival against *P. aeruginosa*–mediated killing. Seven collagen genes (*col-80*, *col-93*, *col-98*, *col-101*, *col-103*, *col-160*, and *col-179*) were individually targeted. To examine the effectiveness of the RNAi of these collagen genes, we performed qRT-PCR to measure the mRNA levels of the targeted genes. Compared to the control RNAi with an empty vector, RNAi of the collagen genes knocked down their expression by 40 to 70% in both wild-type and *npr-8(ok1439)* animals (fig. S4A). RNAi of *col-80* and *col-98* fully suppressed the enhanced survival of *npr-8(ok1439)* animals to the wild-type level (Fig. 2A); RNAi of *col-93*, *col-103*, and *col-160* partially suppressed their enhanced survival (Fig. 2A); and RNAi of *col-101* and *col-179* strongly suppressed the mutants’ survival to levels that were even lower than the wild-type level (Fig. 2A). RNAi of *col-101* and *col-*179 also had an inhibitory effect on the survival of wild-type animals (Fig. 2B). These results indicate that collagen genes are required for *C. elegans* defense against *P. aeruginosa* infection and that their up-regulation in *npr-8(ok1439)* animals contributes to the mutant’s improved survival. To investigate whether RNAi of collagen genes affects overall life span, *col-101* and *col-179* were inactivated by RNAi in wild-type and *npr-8(ok1439)* animals followed by life-span assays on heat-killed *E. coli* strain OP50. Results showed that RNAi of neither gene had any effect on the life span of the mutants, but for wild-type animals, RNAi of *col-101* shortened their life span, while RNAi of *col-179* had no effect (fig. S4, B and C). These results indicate that *col-101* could play a role in maintaining *C. elegans* life span. Given this possible role of *col-101*, it is surprising to observe that *col-101* RNAi had no effect on the mutants. A plausible explanation would be that because a number of collagen genes are induced in the mutants under nonpathogenic conditions (table S2C), one or more of these genes could redundantly function with *col-101* in life-span maintenance, thus negating the effect of *col-101* RNAi.

The defense activity of collagens was further investigated using transgenic animals with deletion mutations in collagen genes [*col-80 (sun1)*, *col-98 (sun2)*, and *col-179(ok3010)* null animals]. Survival assays against *P. aeruginosa* showed that deletion of *col-80* or *col-98* did not alter the survival of wild-type animals but fully suppressed the enhanced survival of *npr-8(ok1439)* animals (Fig. 2, C and D). No additive survival disadvantage was observed in double mutants *npr-8(ok1439);col-80(sun1)* and *npr-8(ok1439);col-98(sun2)* compared to single mutants *col-80(sun1)* and *col-98(sun2)*, respectively (Fig. 2, C and D), indicating that NPR-8 and these collagens likely function in the same defense pathway against *P. aeruginosa* infection. Deletion of *col-179*, however, significantly suppressed the survival of both wild-type and *npr-8(ok1439)* animals (Fig. 2E), indicating an important and larger role of this gene in the defense against infection. Double mutant *npr-8(ok1439);col-179(ok3010)* exhibited no further survival disadvantage compared to single mutant *col-179(ok3010)*, which indicates that COL-179 likely functions downstream of NPR-8 in a linear pathway in defense (Fig. 2E). These results are consistent with the above-described RNAi experiments, both of which indicate that collagen genes are required for *C. elegans* defense against *P. aeruginosa* infection and contribute to the improved survival of *npr-8(ok1439)* animals.

Because up-regulation of collagen genes in *npr-8(ok1439)* animals leads to the mutants’ enhanced survival, we asked whether collagen gene overexpression in wild-type animals can mimic this phenotype. To this end, we generated transgenic strain JRS22 that overexpresses *col-179* mRNA (fig. S4D). Survival assays against *P. aeruginosa* showed that JRS22 animals died at a rate similar to wild-type animals, while *npr-8(ok1439)* animals died significantly more slowly than either JRS22 or wild-type animals (fig. S4E). These results indicate that unlike *npr-8* mutations that enhance survival against infection, *col-179* overexpression does not provide survival advantage, even though *col-179* is overexpressed in *npr-8(ok1439)* animals. A plausible explanation for this discrepancy is that besides *col-179*, a number of other collagen genes are also induced in *npr-8(ok1439)* animals (Table 1B); the combined effect of these induced collagens could change the cuticle structure, which, in turn, enhances the animals’ survival; and this combined effect cannot be mimicked by overexpressing *col-179* alone.

To determine whether the defense activity of collagen genes is specific to those up-regulated in *npr-8(ok1439)* animals under pathogenic conditions, we tested six more collagen genes for their contributions to the improved survival of the *npr-8* mutant animals. From our RNA-seq data (GEO accession number GSE122544), we randomly chose three collagen genes whose expressions were not affected by either *P. aeruginosa* infection or the *npr-8* mutation as unrelated control collagen genes. These are *col-64*, *col-76*, and *col-116*. We also selected three collagen genes—*col-7*, *col-39*, and *col-167*—that were enriched in *npr-8(ok1439)* animals relative to wild-type animals under nonpathogenic conditions but not enriched under infectious conditions. The above six genes were individually inactivated by RNAi in wild-type and *npr-8(ok1439)* animals followed by survival assays against *P. aeruginosa*–mediated killing. RNAi of these genes had no effects on the survival of either wild-type or the mutant animals (fig. S4, F to K). This contrasts with the RNAi of the collagen genes that were enriched under infectious conditions (Fig. 2, A and B). Although only a small number of collagen genes were examined, our results support the idea that the defense activity of collagen genes could be specific to those induced by infection in *npr-8(ok1439)* animals relative to wild-type animals.

We have demonstrated that the *npr-8* null mutation causes faster defecation (Fig. 1F). We asked whether changes in collagens are also responsible for the phenotype of increased defecation rate. To answer this question, we examined how the lack of NPR-8–regulated collagens affects *C. elegans* defecation. The defecation rates of three collagen-null mutants [*col-80(sun1)*, *col-98(sun2)*, and *col-179(ok3010)* null animals] were measured and compared to those of wild-type and *npr-8*(*ok1439)* animals. Among the three collagen mutants, *col-80(sun1)* and *col-179(ok3010)* animals showed a defecation rate similar to the wild-type level, whereas *col-98(sun2)* animals defecated at a significantly faster rate than wild-type animals, a phenotype also displayed by *npr-8(ok1439)* animals (fig. S4L). These results indicate that NPR-8 could control defecation by regulating collagens, although not all NPR-8–regulated collagens are involved in this process. It is likely that NPR-8 controls defecation and defense independently by regulating different subsets of collagens.

### NPR-8 regulates the dynamics of cuticle structure in response to pathogen infection

NPR-8–regulated collagen genes are cuticular by bioinformatics terms (*28*). It is unclear where they are actually expressed or function in defense. To determine the location of the collagen gene expression, we generated two transgenic GFP reporter strains to localize the expression of *col-179* and *col-101*, respectively. These two collagen genes have the biggest effects on the survival of *npr-8(ok1439)* animals against infection (Fig. 2A). Fluorescent imaging of transgenic animals *col-179p::col-179gDNA::gfp* and *col-101p::SL2::gfp* revealed that both *col-179* and *col-101* were extensively expressed in the cuticle as well as in hypodermal and rectal cells (fig. S5, A and B). Given the importance of the cuticle and the hypodermis as physical barriers in resisting pathogen infection and the importance of rectal cuticle in controlling defecation behavior (*29*), the observed expression patterns of NPR-8–regulated collagens explain the regulatory roles of NPR-8 in defense and defecation.

**Fig. 3:**
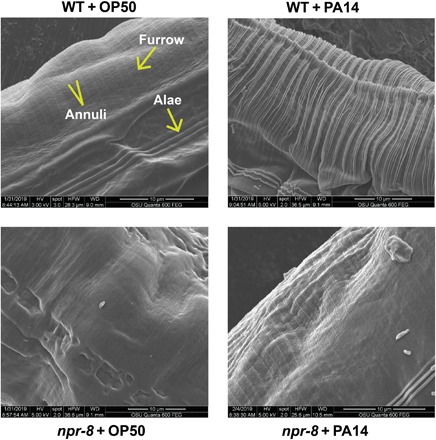
NPR-8 regulates the dynamics of cuticle structure in response to pathogen infection. WT and *npr-8(ok1439)* animals were exposed to *E. coli* OP50 or *P. aeruginosa* PA14 for 24 hours and imaged using FEI Quanta 600 FEG SEM.

The cuticular expression of NPR-8–regulated collagens and their induction in response to infection imply that NPR-8 can function in defense by affecting the cuticle structure. To determine how pathogen infection alters the *C. elegans* cuticle structure and whether the changes, if any, are regulated by NPR-8, we performed scanning electron microscopy (SEM) and transmission electron microscopy (TEM) to examine the cuticle of wild-type and *npr-8(ok1439)* animals with or without 24 hours of exposure to *P. aeruginosa*. Around the body, the cuticle forms small circumferential furrows separated by broader ridges called annuli; this arrangement is interrupted laterally by longitudinally oriented ridges called alae that are generated by cells of the lateral seam beneath (*30*). SEM micrographs showed that upon exposure to *P. aeruginosa* for 24 hours, the cuticle of wild-type young adults changed from smooth to wrinkled looking, like the appearance of older animals (Fig. 3) (*29*). In contrast, the changes in the cuticle of *npr-8(ok1439)* animals were visually less obvious (Fig. 3). To examine the structural changes underneath the surface, we sectioned the animals and performed TEM analyses. Results showed that the thickness of the cuticle in wild-type animals significantly reduced upon exposure to *P. aeruginosa* for 24 hours, whereas the changes in thickness in *npr-8(ok1439)* animals were insignificant (fig. S5, C and D). The above observations indicate that the nematode’s cuticle structure dynamically changes in response to pathogen infection, and the dynamics is NPR-8 dependent. A model emerges whereby a subset of collagen genes is suppressed by NPR-8, which alters the cuticle structure that, in turn, negatively affects the nematode’s defense against infection.

### NPR-8 is expressed in amphid sensory neurons

**Fig. 4:**
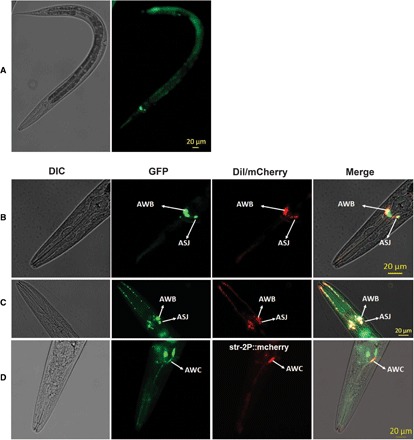
NPR-8 is expressed in amphid sensory neurons. (**A**) The transcriptional reporter strain *npr-8p::gfp* expresses GFP under the control of the *npr-8* native promoter. (**B**) *npr-8p::gfp* expression and DiI dye–filled neurons overlapped in AWB and ASJ neurons (dorsal view). (**C**) *npr-8p::gfp* expression and DiI dye–filled neurons overlapped in AWB and ASJ neurons (ventral view). (**D**) *npr-8p::gfp* expression and *str-2p::mcherry* expression overlapped in AWC neurons (dorsal view). DIC, differential interference contrast microscopy; DiI, DiI staining of amphid neurons; mCherry, mCherry fluorescence microscopy; Merge, overlay of GFP and DiI or mCherry images.

To find out where NPR-8 is expressed in *C. elegans*, we generated a transcriptional reporter strain, *npr-8p::gfp*, that expresses a GFP tag under the control of the 2-kb *npr-8* native promoter. Fluorescent imaging showed prominent expression of GFP in three pairs of neurons close to the pharyngeal bulb in the nematode’s head region and mild fluorescence in the gut region (Fig. 4A). The ability of the *npr-8* mutation to confer resistance to bacterial infection, along with its expression in pharyngeal neurons, prompted us to hypothesize that NPR-8–expressing neurons are sensory in function. To find out the identities of these neurons, we performed DiI [1,1′-dioctadecyl-3,3,3′,3′-tetramethylindocarbocyanine perchlorate] staining, which stains amphid neurons (*31*), on *npr-8p::gfp* animals. Overlapping of DiI staining with GFP fluorescence identified those two pairs of NPR-8–expressing neurons to be AWB and ASJ neurons (Fig. 4B, dorsal view; Fig. 4C, ventral view). Upon analyzing cell position and axon structure, we speculated that the third pair of neurons was AWC neurons. To test this prediction, we generated transgenic animals *npr-8p::gfp,str-2p::mCherry* that coexpress a GFP tag under the *npr-8* promoter and an mCherry tag under the *str-2* promoter, which is specific for driving gene expression in AWC neurons (*32*). Colocalization of GFP fluorescence with mCherry fluorescence confirmed that the third pair of neurons was AWC neurons (Fig. 4D). These results demonstrate that NPR-8 is expressed in amphid sensory neurons. The mild fluorescence in the gut region of *npr-8p::gfp* transgenic animals could be background fluorescence conferred by the backbone of the plasmid pPD95.77 (*33*). However, it could also result from GFP expression driven by the *npr-8* promoter. We generated another reporter strain *npr-8p::mCherry* that expresses an mCherry tag under the control of the *npr-8* promoter using plasmid pHT101 as the backbone (plasmid no. 61021, Addgene). Fluorescent imaging showed that NPR-8 was expressed in AWB, ASJ, and AWC neurons as well as in the gut region (fig. S6A). Because both *npr-8p::gfp* and *npr-8p::mCherry* reporter strains were created with transgenes that use *unc-54* 3′ untranslated region (3′UTR), which could cause false intestinal expression (*33*), it is unclear whether NPR-8 expression in the gut was an artifact of *unc-54* 3′UTR or recapitulated native expression driven by its promoter.

To find out how *npr-8* changes its expression during development, we imaged the expression of *npr-8p::gfp* reporter at different developmental stages, including embryo, L1 larva, L2 larva, L3 larva, L4 larva, and young adult. The images are presented in fig. S6B. In all developmental stages except embryo, expression of GFP was observed in three pairs of head neurons (AWB, AWC, and ASJ) and the lower gut region. Compared to wild-type N2 animals, *npr-8p::gfp* transgenic animals did not exhibit any noticeable changes in growth, size, or behavior.

### The role of NPR-8 in defense response to pathogen infection is confined to neuronal cells

**Fig. 5:**
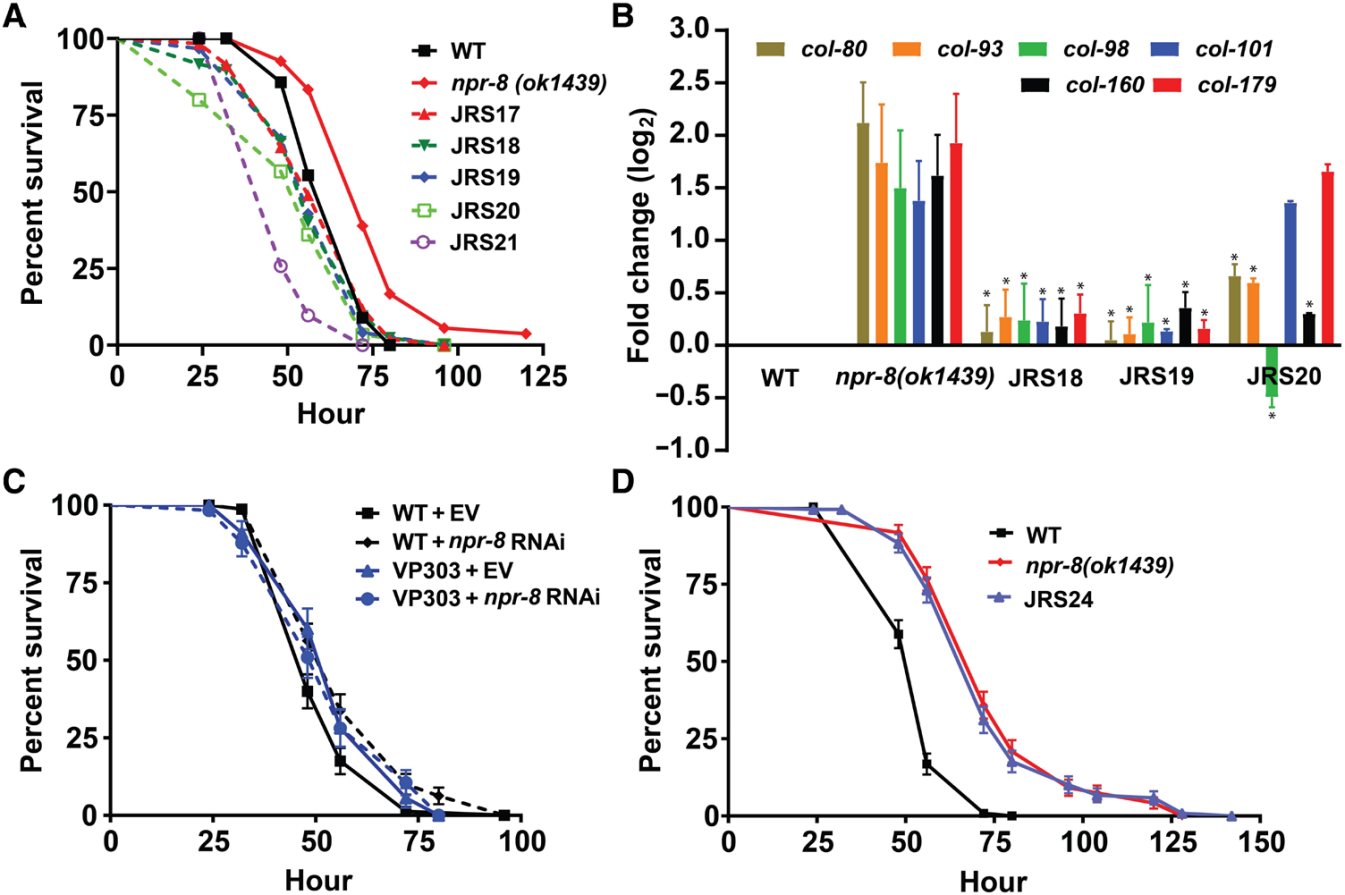
NPR-8 functions in neuronal cells not in the gut to defend *C. elegans* against *P. aeruginosa* infection. (**A**) WT, *npr-8(ok1439)*, and rescue animals were exposed to *P. aeruginosa* and scored for survival over time. JRS17, *npr-8* rescue by its own promoter; JRS18, *npr-8* rescue in AWB neurons; JRS19, *npr-8* rescue in AWC neurons; JRS20, *npr-8* rescue in ASJ neurons; JRS21, *npr-8* pan-neuronal rescue. The graphs are the combined results of three independent experiments. Each experiment included *n* = 60 adult animals per strain. *P* values are relative to *npr-8(ok1439)* animals: WT, *P* < 0.0001; JRS17, *P* < 0.0001; JRS18, *P* < 0.0001; JRS19, *P* < 0.0001; JRS20, *P* < 0.0001; JRS21, *P* < 0.0001. (**B**) qRT-PCR analysis was performed to measure the expression of collagen genes in WT, *npr-8(ok1439)*, JRS18, JRS19, and JRS20 animals exposed to *P. aeruginosa*. In all assays, pan-actin served as internal controls. Bars represent mean ± SEM, and values are the average of two independent experiments. Asterisks (*) denote a significant difference (*P* < 0.0001) in expression of collagen genes between *npr-8(ok1439)* animals and the rescue strains. (**C**) WT and VP303 (for intestine-specific RNAi) animals grown on dsRNA for *npr-8* or EV control were exposed to *P. aeruginosa* and scored for survival over time. The graphs are the combined results of three independent experiments. Each experiment included *n* = 60 adult animals per strain. *P* values are relative to WT + EV: WT + *npr-8* RNAi, *P* = 0.0062; VP303 + vector, *P* = 0.1141; VP303 + *npr-8* RNAi, *P* = 0.2153. (**D**) WT, *npr-8(ok1439)*, and JRS24 animals were exposed to *P. aeruginosa* and scored for survival over time. JRS24, *npr-8* rescue in the intestine. The graphs are the combined results of two independent experiments. Each experiment included *n* = 60 adult animals per strain. WT versus *npr-8(ok1439)*, *P* < 0.0001; WT versus JRS24, *P* < 0.0001; *npr-8(ok1439)* versus JRS24, *P* = 0.6979.

To determine the activity foci of NPR-8 in *C. elegans* defense against pathogen infection, we generated rescue strains to restore NPR-8 expression in different locations of *npr-8(ok1439)* animals and then assayed their survival against *P. aeruginosa*–mediated killing. Restoration of NPR-8 expression under its own promoter rescued the enhanced survival of *npr-8(ok1439)* animals against *P. aeruginosa* (Fig. 5A). Restoration of NPR-8 expression under a pan-neuronal promoter or cell-specific promoters for either AWB, ASJ, or AWC neurons fully rescued the enhanced survival of the mutant animals (Fig. 5A). These results indicate that NPR-8 functions redundantly in the three different neuron pairs to regulate *C. elegans* defense against *P. aeruginosa* infection.

Next, we investigated whether NPR-8 expression in the individual neurons AWB, ASJ, or AWC rescues the collagen overexpression phenotype of *npr-8(ok1439)* animals. To this end, we performed qRT-PCR experiments to measure the mRNA levels of six collagen genes (*col-80*, *col-93*, *col-98*, *col-101*, *col-160*, and *col-179*) in wild-type, *npr-8(ok1439)*, and the three rescue strains. As expected, these genes were significantly up-regulated in *npr-8(ok1439)* relative to wild-type animals exposed to *P. aeruginosa* (Fig. 5B). In the rescue animals, however, their expressions were significantly suppressed when compared to *npr-8(ok1439)* animals, except *col-101* and *col-179* in strain JRS20 (*npr-8* rescued in ASJ neurons) that remained relatively unchanged (Fig. 5B). These results indicate that NPR-8 functions redundantly in AWB, AWC, and ASJ neurons to regulate collagen expression in response to infection.

To investigate whether NPR-8 functions in the gut in the defense against pathogen infection, we used tissue-specific RNAi to inhibit *npr-8* activity, if any, in the intestine and then assayed the nematode’s survival against *P. aeruginosa*–mediated killing. Transgenic strain VP303 (*34*) that is capable of tissue-specific RNAi in the intestine was used. Survival assays showed that inactivation of *npr-8* in the intestine had no effects on the enhanced survival of *npr-8(ok1439)* animals following exposure to *P. aeruginosa* (Fig. 5C). In a separate experiment, we rescued *npr-8* expression in the gut of *npr-8(ok1439)* animals under the 2.1-kb *ges-1* promoter (*23*). The resulting rescue strain JRS24 along with wild-type and *npr-8(ok1439)* animals were subject to survival assays against *P. aeruginosa*–mediated killing. Results showed that *npr-8* expression in the gut did not rescue the enhanced survival phenotype of *npr-8(ok1439)* animals (Fig. 5D). These results, combined with the NPR-8 restoration experiments, indicate that NPR-8 functions in neurons, not in the gut, to regulate *C. elegans* defense against *P. aeruginosa* infection.

### NPR-8 is not involved in sensing bacteria

Expression of NPR-8 in sensory neurons, particularly in AWB and AWC neurons that are important for sensing olfactory stimuli (*35*), raised the question about NPR-8’s role in chemosensation. *C. elegans* exhibits food choice behavioral changes through its ability to sense olfactory stimuli by sensory neurons (*35*). To determine whether NPR-8 plays a role in olfaction, we performed food choice assays in which animals were placed on plates containing spots of two types of bacteria, *E. coli* OP50 and *P. aeruginosa* PA14; then, at later times, the distributions of the animals on these bacteria were counted and calculated to give a choice index [CI = (number of animals on *P. aeruginosa* − number of animals on *E. coli*)/total number of animals; a positive value means preference for *P. aeruginosa* and a negative value means preference for *E. coli*] (*36*). Our results showed that the CIs of *npr-8(ok1439)* animals were similar to those of wild-type animals at the two time points tested (4 and 24 hours), all of which were negative (fig. S7), indicating that both the mutant and wild-type animals preferred *E. coli* over *P. aeruginosa* as food to a similar degree. This result is consistent with the *P. aeruginosa* lawn avoidance (fig. S1G) and lawn occupancy assays (Fig. 1C), showing that the mutant animals displayed pathogen avoidance behavior similar to wild-type animals. Together, these experiments suggest that *npr-8* does not function in sensing bacterial olfactory stimuli.

## DISCUSSION

In the current study, we have characterized the roles of NPR-8 in *C. elegans* defense against pathogen infection. We have found that functional loss of NPR-8 enhances the nematode’s survival against the three pathogens tested (*P. aeruginosa*, *S. enterica*, and *S. aureus*). This enhanced survival is not due to pathogen avoidance behavior or reduced oral uptake of pathogens; however, increased pathogen clearance from the intestine and faster defecation were observed in *npr-8* mutants. RNA-seq analyses revealed that the expression of cuticular collagen genes was enriched in *npr-8* mutants compared to wild-type animals under both uninfected and infected conditions. Subsequently, we showed that regulation of collagens by NPR-8 is essential for the nematode’s defense against infection, and the dynamics of cuticle structure in response to infection is NPR-8 dependent. We further demonstrated that the NPR-8 defense activity is confined to amphid sensory neurons AWB, ASJ, and AWC; however, it is not involved in the bacteria-sensing functions of these neurons. This suggests that the dynamics of cuticle structure in response to pathogen infection is regulated by the nervous system. It is generally believed that physical barrier defenses are not a response to infections; they are part of the body’s basic innate defense and are continuously working to protect against a broad range of pathogens. Our results challenge this view by demonstrating not only that *C. elegans* cuticle structure dynamically changes in response to pathogen infection but also that the cuticle barrier defense is regulated by the nervous system. It remains unknown how NPR-8–expressing neurons in the head of *C. elegans* regulate the defense response in the cuticle. We speculate that this regulation is achieved through a neuroendocrine signaling pathway. The non–cell-autonomous signaling could be mediated by neurotransmitters, neuropeptides, and/or neurohormones and will be the subject of our future investigation.

Pathogen infection–induced collagen expression has also been observed in other studies (*37*, *38*). For example, Wong *et al.* (*37*) showed that up-regulation of *C. elegans* cuticular collagens was part of a shared response to multiple pathogens (*Serratia marcescens*, *Enterococcus faecalis*, *Erwinia carotovora*, and *Photorhabdus luminescens*). Why would *C. elegans* change its cuticle structure in response to infections? Some bacterial species secrete extracellular proteases that can degrade collagen molecules, resulting in a weakened hypodermis that allows bacteria or bacterial molecules to enter the host (*39*, *40*). Understandably, *C. elegans* would up-regulate cuticular collagens to counter these effects. Our SEM micrographs showed that upon exposure to *P. aeruginosa* for 24 hours, the cuticle of wild-type young adults changed from smooth to wrinkled looking (Fig. 3). Cuticle wrinkles are an indication of a weaker, thinner hypodermis or loosened connections between the cuticle and hypodermis (*29*). This implies that the cuticle structure has changed because of *P. aeruginosa* attack, and the nematode was not able to effectively counter this effect at the time point examined. *npr-8(ok1439)* animals, however, displayed less-wrinkled cuticle at the same infection time point, suggesting that the *npr-8* mutants somewhat remedied the damage inflicted by *P. aeruginosa*. Combined with the fact that *npr-8* mutants have higher levels of expression of cuticular collagens and survive better than wild-type animals, a model emerges whereby NPR-8 regulates the cuticle dynamics by suppressing the expression of cuticular collagen genes, which, in turn, negatively affects the nematode’s defense against infection. It seems paradoxical that wild-type animals have a negative regulatory mechanism to limit their cuticle barrier defense. However, the cuticle is a multifunctional exoskeleton. On the one hand, it serves as a barrier between the nematode and pathogens or other environmental assaults; on the other hand, it allows the nematode to absorb chemical cues or nutrients from the environment (*41*). Therefore, we speculate that the NPR-8–mediated neural cuticle structure regulatory circuit is necessary to maintain homeostasis between the nematode and its environment.

In the current study, we observed that *npr-8(ok1439)* animals defecate faster than wild-type animals and showed that inactivation of the *C. elegans* DMP did not rescue the enhanced survival phenotype of *npr-8* mutant animals, indicating that faster defecation and enhanced survival are two independent phenotypes caused by the *npr-8* mutation. Rescuing *npr-8* expression in two defecation-regulating neurons, AVL and DVB, in *npr-8(ok1439)* animals significantly suppressed the mutants’ increased defecation rate but did not suppress their enhanced survival, confirming that faster defecation and enhanced survival are two independent phenotypes. This rescue experiment revealed that NPR-8 likely functions in AVL and DVB neurons to control defecation. One caveat for this conclusion is that NPR-8 expression in these neurons was not observed in the *npr-8p::gfp* reporter strain or the *npr-8p::mCherry* reporter strain (Fig. 4A and fig. S6A). It is possible that the levels of NPR-8 expression in these neurons are too low to be seen in the reporter strains. We have already shown that NPR-8 acts in amphid sensory neurons AWB, ASJ, and AWC to regulate *C. elegans* defense (Fig. 5A). These data indicate that NPR-8, a single type of neuronal GPCR, may function in two distinct neural circuits to regulate defecation and defense, respectively. *col-98(sun2)* animals, but not *col-80(sun1)* or *col-179(ok3010)* animals, exhibited the faster defecation phenotype (fig. S4L), indicating that NPR-8 could control defecation by regulating a subset of collagens that are different from the subset it regulates in defense. How do changes in collagens affect defecation? Although the underlying molecular mechanisms are currently unclear, it has been observed that, in older worms, thickened rectal cuticle limits motility, leading to a decline in defecation with age (*29*). This observation indicates that collagens could modulate defecation behavior through their structural role in rectal cuticle. Finding out which collagen genes besides *col-98* are involved in modulating defecation behavior and how NPR-8 regulates this process would be interesting topics for our future studies.

Cuticular collagens have been implicated in *C. elegans* resistance to oxidative stress and longevity in general (*42*). The expression levels of particular collagen genes decline with age; increased collagen expression is a shared feature of multiple conserved longevity pathways and of essentially every longevity intervention (*42*, *43*). Our results support an important role of collagens in life span under the stress of pathogen infection. It is possible that NPR-8–mediated neural regulation of collagens is important for host defense against other environmental assaults and might play a role in general longevity control.

## MATERIALS AND METHODS

### Experimental design

Our objective was to characterize the roles of NPR-8 in *C. elegans* defense against pathogen infection. In the current study, we addressed three questions. First, does NPR-8 play a role in *C. elegans* defense against infections? Second, what is the molecular basis for NPR-8’s role, if any? Third, where are the activity foci of NPR-8 in *C. elegans*? To answer these questions, we determined how functional loss of NPR-8 in *npr-8(ok1439)* null animals affected their survival against various pathogenic bacteria. Our results showed that the mutant animals displayed enhanced survival against all three pathogens tested. We then examined the animals’ pathogen avoidance behavior, pathogen intake and accumulation, and innate immunity to determine whether any of these factors contribute to the mutants’ altered survival. To gain molecular insights into how NPR-8 regulates *C. elegans* defense, we used RNA-seq to profile gene expression in *npr-8(ok1439)* animals relative to wild-type animals with or without pathogen infection. To identify the foci of activity of NPR-8, we generated reporter strains to map NPR-8 expression pattern and transgenic strains to rescue *npr-8(ok1439)* animals using cell- and tissue-specific *npr-8* transgenes.

### Bacterial strains

The following bacterial strains were used: *E. coli* OP50, *P. aeruginosa* PA14, *S. enterica* serovar Typhimurium SL1344, and *S. aureus* MSSA476. These bacteria were grown in Luria-Bertani (LB) broth at 37°C. *P. aeruginosa* expressing GFP was grown in LB medium supplemented with ampicillin (100 μg/ml).

### *C. elegans* strains

*C. elegans* strains were cultured under standard conditions and fed *E. coli* OP50. Wild-type animals were *C. elegans* Bristol N2. RB1321 *npr-8(ok1439)*, RB1329 *npr-8(ok1446)*, RB2225 *col-179(ok3010)*, and VP303 *rde-1(ne219); kbIs7[pnhx-2::rde-1, rol-6]* animals were obtained from the *Caenorhabditis elegans* Genetics Center (University of Minnesota, Minneapolis, MN). Transgenic strains JRS13, JRS14, JRS17, JRS18, JRS19, JRS20, JRS21, JRS22, JRS24, JRS25, JRS26, JRS30, JRS37, JRS38, JRS39, and JRS40 were generated in this study; genetic details of these strains are listed in table S3.

### *C. elegans* survival assay

Wild-type worms and mutants were maintained as hermaphrodites at 20°C and fed *E. coli* OP50 on modified nematode growth medium (NGM) agar plates (0.35% instead of 0.25% peptone) (*44*). The bacterial lawn used for *C. elegans* killing assays was prepared by spreading a 25-μl drop of an overnight culture of bacteria on modified NGM agar plates (3.5-cm-diameter petri plates). Plates were incubated at 37°C for 16 hours and then cooled down at room temperature for at least 1 hour before seeding with synchronized worms. Survival assays were performed at 25°C, and live worms were transferred daily to fresh plates. Worms were scored at the times indicated and were considered dead when they failed to respond to touch.

### Bacterial lawn avoidance assay

Bacterial lawn avoidance assays were performed as described (*10*). Briefly, small lawns of *P. aeruginosa* PA14 were cultured on 3.5-cm NGM plates at 37°C overnight. Twenty young adult animals grown on *E. coli* OP50 were transferred to the center of each bacterial lawn. The number of animals on the bacterial lawns was counted at 4, 8, 12, 24, and 48 hours after exposure.

### Pharyngeal pumping rate assay

Wild-type and *npr-8(ok1439)* animals were synchronized by placing 20 gravid adult worms on NGM plates seeded with *E. coli* OP50 and allowing them to lay eggs for 45 min at 25°C. The gravid adult worms were then removed, and the eggs were allowed to hatch and grow at 20°C until they reached the young adult stage. The synchronized worms were transferred to NGM plates fully seeded with *P. aeruginosa* for 24 hours at 25°C. Worms were observed under the Leica M80 microscope with focus on the pharynx. The number of contractions of the pharyngeal bulb was counted over 30 s. Counting was conducted in triplicate and averaged to give a pumping rate.

### Profiling of bacterial accumulation in the intestine

To determine the profiles of bacterial accumulation in the intestine, synchronized L1 larvae [wild-type and *npr-8(ok1439)*] were grown on *E. coli* OP50 at 20°C until they reached the young adult stage. The animals were then transferred to plates seeded with *P. aeruginosa*/GFP and cultured at 25°C for 24 hours. To eliminate *P. aeruginosa*/GFP bound to the body before microscopy, the animals were transferred to NGM plates seeded with *E. coli* and incubated for 15 min, followed by two more transfers to new NGM plates seeded with *E. coli* with 30-min incubation after each transfer. The animals were then visualized using a Zeiss Axio Imager fluorescence stereomicroscope.

### Quantification of intestinal bacterial loads

To quantify the CFUs of intestinal bacterial loads, synchronized L1 larvae [wild-type and *npr-8(ok1439)*] were grown on *E. coli* OP50 at 20°C until they reached the young adult stage. The animals were then transferred to plates seeded with *P. aeruginosa*/GFP and cultured at 25°C for 24 hours. To eliminate *P. aeruginosa*/GFP bound to the body, the animals were transferred to NGM plates seeded with *E. coli* and incubated for 15 min, followed by one more transfer to new NGM plates seeded with *E. coli* with 30-min incubation. Ten animals per condition were placed into 50-μl phosphate-buffered saline (PBS) with 0.1% Triton and then grounded. Serial dilutions of the lysates (10^−1^, 10^−2^, 10^−3^, 10^−4^) were plated onto LB/kanamycin plates to select for *P. aeruginosa*/GFP cells and incubated at 37°C for 24 hours. The CFUs on the plates were then counted.

### Defecation rate assay

Wild-type and mutant animals were synchronized by placing 20 gravid adult worms on NGM plates seeded with *E. coli* OP50 and allowing them to lay eggs for 45 min at 25°C. The gravid adult worms were then removed, and the eggs were allowed to hatch and grow at 20°C until they reached the young adult stage. The synchronized worms were transferred to NGM plates fully seeded with *P. aeruginosa* for 24 hours at 25°C. Worms were observed under a Leica M80 microscope with a 60× magnification at room temperature. For each worm, an average of 10 intervals between two defecation cycles were measured. The defecation cycle was identified as a peristaltic contraction beginning at the posterior body of the animal and propagating anteriorly followed by feces expulsion.

### DiI staining of amphid neurons

Amphid neurons were stained with DiI (D-282, Molecular Probes) as described (*45*) with slight modifications. Briefly, well-fed young adult animals were collected from 6-cm plates, washed twice with M9 buffer, and incubated in 1 ml of M9 buffer containing 5 μl of DiI [dilution of 1:200 of stock (2 mg/ml)] overnight at room temperature. After incubation, the animals were washed three times with M9 buffer and transferred to NGM plates seeded with *E. coli* OP50. After incubating for 1 hour, the animals were mounted on agarose pads with 50 mM sodium azide and imaged using a Zeiss M2 microscope with a mechanized stage, Zen software, and Texas red filters.

### Food choice assay

Assays were performed in 6-cm NGM plates. Five-microliter bacterial cultures (*E. coli* OP50 or *P. aeruginosa* PA14) were spotted at equal distances from the center of the plate and incubated at 37°C for 12 to 16 hours. Synchronized young adult animals [wild-type and *npr-8(ok1439)*] were placed at the center of the plate. At later times (4 and 24 hours), animals on each bacterial spot were scored and calculated to give a CI [CI = (number of animals on *P. aeruginosa* − number of animals on *E. coli*)/total number of animals] (*36*). A positive CI indicates preference for *P. aeruginosa*; a negative CI indicates preference for *E. coli*; and an index of zero indicates no preference between the two bacteria.

### RNA isolation

Gravid adult animals were lysed using a solution consisting of sodium hydroxide and bleach at a volume ratio of 5:2. The eggs were washed and synchronized for 22 hours in S basal liquid medium at room temperature. Synchronized L1 larval animals were placed onto NGM plates seeded with *E. coli* OP50 and grown at 20°C until the animals reached L4 stage. Animals were collected, washed with M9 buffer, transferred to NGM plates containing *P. aeruginosa* PA14, and then incubated for 4 hours at 25°C. The animals were then collected and washed with M9 buffer, and RNA was extracted using QIAzol lysis reagent (Qiagen) and purified with the RNeasy Plus Universal Kit (Qiagen).

### Quantitative real-time PCR

Total RNA was obtained as described above and subjected to reverse transcription using the High-Capacity cDNA Reverse Transcription Kit (Applied Biosystems). Quantitative PCR with 50 ng of complementary DNA (cDNA) per 25 μl of reaction was conducted on a StepOnePlus Real-Time PCR system using Power SYBR Green PCR Master Mix in a 96-well plate format (Applied Biosystems). Relative fold changes for transcripts were calculated using the comparative *C*_T_ (2^−ΔΔ*C*^_T_) method and normalized to pan-actin (*act-1, act-3,* and *act-4*). Cycle thresholds of amplification were determined by StepOne Software v2.3 (Applied Biosystems). All samples were run in triplicate. Primer sequences are available upon request.

### RNA sequencing

Five replicates of four groups of RNA samples [*npr-8(ok1439)*] and wild-type animals with or without exposure to *P. aeruginosa* for 24 hours were collected and submitted to the WSU Genomics Core for RNA-seq analyses. The integrity of total RNA was assessed using Fragment Analyzer (Advanced Analytical Technologies, Ankeny, IA) with the High Sensitivity RNA Analysis Kit. RNA quality numbers (RQNs) from 1 to 10 were assigned to each sample to indicate its integrity or quality. “10” stands for a perfect RNA sample without any degradation, whereas “1” marks a completely degraded sample. RNA samples with RQNs ranging from 8 to 10 were used for RNA library preparation with the TruSeq Stranded mRNA Library Prep Kit (Illumina, San Diego, CA). Briefly, mRNA was isolated from 2.5 μg of total RNA using poly-T oligo attached to magnetic beads and then subjected to fragmentation, followed by cDNA synthesis, dA-tailing, adaptor ligation, and PCR enrichment. The sizes of the RNA libraries were assessed using Fragment Analyzer with the High Sensitivity NGS Fragment Analysis Kit. The concentrations of the RNA libraries were measured using the StepOnePlus Real-Time PCR System (Thermo Fisher Scientific, San Jose, CA) with the KAPA Library Quantification Kit (Kapa Biosystems, Wilmington, MA). The libraries were diluted to 2 nM with a resuspension buffer (10 mM tris-HCl, pH 8.5) and denatured with 0.1 N NaOH. Libraries (18 pM) were clustered in a high-output flow cell using HiSeq Cluster Kit v4 on a cBot (Illumina). After cluster generation, the flow cell was loaded onto a HiSeq 2500 instrument for sequencing using HiSeq SBS Kit v4 (Illumina). DNA was sequenced from both ends (paired end) with a read length of 100 base pairs (bp). The raw BCL files were converted to FASTQ files using the software program bcl2fastq2.17.1.14. Adaptors were trimmed from the FASTQ files during the conversion. On average, 40 million reads with a read length of 2 × 100 bp were generated for each sample.

RNA-seq data (FASTQ files) were aligned to the *C. elegans* reference genome (ce10, UCSC) using HISAT2 (*46*). Gene expression quantification and differential expression were analyzed using HTSeq and DESeq2 (*47*). The sequencing data (FASTQ files) have been deposited in NCBI’s SRA database through the GEO; processed gene quantification files and differential expression files have been deposited in the GEO; and all of these data can be accessed through the GEO with the accession number GSE122544. GO enrichment analyses were conducted to identify significantly enriched biological processes and molecular functions using the web-based program Gorilla (http://cbl-gorilla.cs.technion.ac.il/) (*48*).

### RNA interference

RNAi was conducted by feeding *C. elegans E. coli* strain HT115(DE3) expressing dsRNA that is homologous to a target gene (*20*). Briefly, *E. coli* with the appropriate vectors was grown in LB broth containing ampicillin (100 μg/ml) at 37°C overnight and plated onto NGM plates containing ampicillin (100 μg/ml) and 3 mM isopropyl-β-d-thiogalactopyranoside (IPTG). RNAi-expressing bacteria were allowed to grow overnight at 37°C. L2 or L3 larval animals were placed on RNAi or vector control plates for 2 days at 20°C until the nematodes became gravid. Gravid adults were then transferred to fresh RNAi-expressing bacterial lawns and allowed to lay eggs for 1 hour to synchronize the second-generation RNAi population. The gravid adults were removed, and eggs were allowed to develop at 20°C to reach young adult stage for subsequent assays. Clone identity was confirmed by sequencing. *unc-22* RNAi was included as a positive control in all experiments to account for RNAi efficiency.

### Plasmid construction and transgenic animal generation

Two kilobases of the *npr-8* promoter was amplified by PCR from the genomic DNA of wild-type *C. elegans* at mixed stages and cloned into vector pPD95.77 (Fire Lab *C. elegans* Vector Kit, Addgene, Cambridge, MA) via the Pst I and Bam HI sites. This resulted in plasmid pSD01. This plasmid contains *unc-54* 3′UTR inherited from pPD95.77. Transcriptional reporter strain JRS13 was generated by injecting pSD01 (50 ng/μl) with *unc-122p::DsRed* (10 ng/μl) as a coinjection marker.

Two-kilobase *npr-8* promoter was spliced from pSD01 and cloned into vector pHT101 (pHT101-mCherry, plasmid no. 61021, Addgene) via the Pst I and Bam HI sites. This resulted in plasmid pSD02. This plasmid contains *unc-54* 3′UTR region inherited from pHT101. Transcriptional reporter strain JRS14 was generated by injecting pSD02 (5 ng/μl) with *unc-122p::gfp* (10 ng/μl) as a coinjection marker. The total concentration of injection mix was made up to 100 ng/μl using plasmid blue script.

The *npr-8* genomic fragment contains nine exons with a large first intron spanning 8581 bp; hence, the *npr-8* genomic rescue plasmid pSDG04 was constructed by fusing two fragments of *npr-8* genomic DNA. The first fragment contained exon 1 of *npr-8* along with the 1000-bp first intron with a Bam HI restriction site at 3′ end and a Sal I site at 5′ end. The second fragment contained exons 2 to 9 with all intron sequences with a Sal I site at 3′ end and an Age I site at 5′ end. Fragments 1 and 2 were fused together at the Sal I site. The resulting genomic fragment was cloned into plasmid pSD01 between Bam HI and Age I to generate rescue plasmid pSDG04. Translational rescue strain JRS17 was generated by injecting plasmid pSDG04 (45 ng/μl) with *unc-122p::gfp* (10 ng/μl) as a coinjection marker.

The *str-1* promoter was amplified from plasmid pTM139 and cloned into pSDG04 between the Pst I and Bam HI sites to generate pSDG05. AWB neuron–specific rescue strain JRS18 was generated by injecting plasmid pSDG05 (75 ng/μl) with *unc-122p::gfp* (10 ng/μl) as a coinjection marker.

The *str-2* promoter was amplified from plasmid pBL207 and cloned into pSDG04 between the Pst I and Bam HI sites to generate pSDG06. AWC neuron–specific rescue strain JRS19 was generated by injecting plasmid pSDG06 (75 ng/μl) with *unc-122p::gfp* (10 ng/μl) as a coinjection marker.

The *trx-1* promoter was amplified from plasmid pJDM160 and cloned into pSDG04 between the Pst I and Bam HI sites to generate pSDG07. ASJ neuron–specific rescue strain JRS20 was generated by injecting plasmid pSDG07 (100 ng/μl) with *unc-122p::gfp* (10 ng/μl) as a coinjection marker.

The *rgef-1* promoter was amplified from plasmid pCB101 and cloned into pSDG04 between the Pst I and Bam HI sites to generate pSDG08. Pan-neuronal rescue strain JRS21 was generated by injecting plasmid pSDG08 (75 ng/μl) with *unc-122p::gfp* (10 ng/μl) as a coinjection marker.

Genomic region containing *col-179* promoter (965 bp) and its coding region including introns (901 bp) was PCR-amplified from the genomic DNA of wild-type *C. elegans* at mixed stages and cloned into pPD95.77 between Pst I and Xma I sites to generate plasmid pSDG09. Translation reporter strain JRS22 was generated by injecting wild-type animals with plasmid pSDG09 (30 ng/μl) with *unc-122::DsRed* (10 ng/μl) as a coinjection marker. Strain JRS22 also served as a *col-179* overexpression strain.

Two kilobases of the *aex-2* promoter was PCR-amplified from the genomic DNA of wild-type *C. elegans* at mixed stages and cloned into plasmid pSDG04 to generate pSDG11. The rescue strain JRS25 was generated by injecting plasmid pSDG11 (30 ng/μl) with pCFJ104 (10 ng/μl) as a coinjection marker (*pCFJ104-Pmyo-3::mCherry::unc-54*, plasmid no. 19328, Addgene).

To localize *col-101* expression, *col-101* promoter region (2.1 kb) was PCR-amplified from the genomic DNA of wild-type *C. elegans* at mixed stages and cloned into pPD95.77 between Pst I and Xma I sites to generate plasmid pSD03. Translation reporter strain JRS26 was generated by injecting wild-type animals with pSD03 (30 ng/μl) with *unc-122::DsRed* (10 ng/μl) as a coinjection marker.

To rescue NPR-8 in intestine, *ges-1* promoter region (2148 bp) was PCR-amplified from the genomic DNA of wild-type *C. elegans* at mixed stages and cloned into pSDG04 to generate pSDG10. Intestine-specific rescue strain JRS24 was generated by injecting *npr-8(ok1439)* animals with pSDG10 (30 ng/μl) with *unc-122::DsRed* (10 ng/μl) as a coinjection marker.

All transgenic strains generated in this study are listed in table S3. The sequences of all genes and promoters were verified by sequencing at Eton Bioscience (Research Triangle Park, NC). The expression patterns of neuron-specific promoters were confirmed for the relevant transgenic strains by microscopic analyses. The strains and plasmids constructed by us and primer sequences used for their construction are available upon request.

### CRISPR genome editing

Transgenic knockout (KO) strains were constructed using the CRISPR-sdm approach (*49*). In brief, an injection mix containing the standard CRISPR components [single-guide RNA (sgRNAs), donor homology (ODN), Cas9 protein, and a co-CRISPR selection tool (dpy-10 sgRNA and dpy-10 ODN)] were microinjected into wild-type animals. The F1 generation animals were screened for the presence of the co-CRISPR phenotype (this phenotype will not be present in the final strain). Deletion of the selected genomic region was confirmed using PCR. Animals positive for the KO were homozygosed, and the homozygous KO was further confirmed by PCR and sequencing. Strain JRS37 carrying KO in *col-80* was generated by precise deletion of 1171 bp spanning exons 1 and 2 and insertion of a three-frame stop codon. Strain JRS38 carrying KO in *col-98* was generated by precise deletion of 1203 bp spanning exons 1, 2, and 3 and insertion of a three-frame stop codon. More strain details are available upon request.

### Electron microscopy analysis

To determine the structural changes in the cuticle, synchronized L1 larvae [wild-type and *npr-8(ok1439)* animals] were grown on *E. coli* OP50 at 20°C until they reached the young adult stage. The animals were then transferred to plates seeded with *E. coli* OP50 or *P. aeruginosa* PA14 and cultured at 25°C for 24 hours. Animals were collected and washed with M9 buffer and incubated in the fixation buffer (2.5% glutaraldehyde, 1.0% paraformaldehyde, and 0.1 M sodium cacodylate buffer). For SEM analysis (*50*), sample suspensions were placed on 0.4-μm Nuclepore filters and dehydrated in graded series of ethanol (10 to 100%) with 10 to 15 min at each grade. The filters were then placed on SEM stubs, sputter-coated with gold:palladium (40:60), and imaged with FEI Quanta 600 FEG SEM. For TEM analysis (*50*), samples were rinsed with 0.1 M sodium cacodylate buffer and fixed with 1.5% potassium ferrocyanide and 2% osmium tetroxide. The samples were then subjected to T-O-T-O staining followed by dehydration in a graded series of acetone (10 to 100%) with 10 to 15 min at each grade. Samples were infiltrated with Araldite resin, ultrathin-sectioned, and placed on naked gold grids. Images were taken with a FEI Helios NanoLab 650 SEM in STEM mode. For each observation, 20 to 25 cross sections from the midbody region were evaluated, and representative images were presented.

### Fluorescence imaging

Fluorescence microscopy imaging of *C. elegans* was performed using a Zeiss Axio Imager M2 fluorescence stereomicroscope equipped with DIC and Zen 2 capture software. Before imaging, animals were immobilized using 30 mM sodium azide on freshly prepared 2% agarose pad and coverslipped with a 1-mm cover glass.

### Statistical analysis

For *C. elegans* survival assays, animal survival was plotted as a nonlinear regression curve using the PRISM computer program (version 6, GraphPad Software Inc., La Jolla, CA). Survival curves were considered different than the appropriate control when *P* values were <0.05. Prism uses the product limit or Kaplan-Meier method to calculate survival fractions and the log-rank test (equivalent to the Mantel-Heanszel test) to compare survival curves. A two-sample *t* test for independent samples was used to analyze qRT-PCR results; *P* values of <0.05 are considered significant. All the experiments were repeated at least three times, unless otherwise indicated.

## Supplementary Material

http://advances.sciencemag.org/cgi/content/full/5/11/eaaw4717/DC1

Download PDF

Neuronal GPCR NPR-8 regulates C. elegans defense against pathogen infection

## SUPPLEMENTARY MATERIALS

Supplementary material for this article is available at http://advances.sciencemag.org/cgi/content/full/5/11/eaaw4717/DC1 Fig. S1. Functional loss of NPR-8 enhances *C. elegans* survival against pathogen infection and increases pathogen clearance from the intestine. Fig. S2. NPR-8 functions in AVL and DVB neurons to regulate defecation but not survival against infection. Fig. S3. NPR-8 does not play a role in conserved innate immune pathways. Fig. S4. NPR-8–regulated collagen genes are involved in *C. elegans* defense and defecation. Fig. S5. NPR-8 regulates collagen expression in the cuticle and hypodermis and controls the dynamics of cuticle structure in response to infection. Fig. S6. NPR-8 is expressed in amphid sensory neurons and throughout developmental stages. Fig. S7. NPR-8 is not involved in bacteria sensing, as determined by food choice assays. Table S1. Differential expression of genes in conserved innate immune pathways in *npr-8(ok1439)* animals relative to wild-type animals exposed to *P. aeruginosa*. Table S2. Ontology analyses of up-regulated genes in *P. aeruginosa*–infected wild-type animals relative to uninfected controls or in uninfected *npr-8(ok1439)* animals relative to wild-type animals. Table S3. List of transgenic *C. elegans* strains generated in this study.

## Appendix

View/request a protocol for this paper from *Bio-protocol*.
