# Supplementary material for: Neuronal GPCR NPR-8 regulates C. elegans defense against pathogen infection
Source: Sci Adv. 2019 Nov 20;5(11):eaaw4717. doi: 10.1126/sciadv.aaw4717 (PMC6867885; doi:10.1126/sciadv.aaw4717)
Supplement: http://advances.sciencemag.org/cgi/content/full/5/11/eaaw4717/DC1 [file supp_5_11_eaaw4717__index.html]

Science Advances | Science AdvancesAAASSearchScience AdvancesMenu

## Supplementary Materials

**This PDF file includes:**

- Fig. S1. Functional loss of NPR-8 enhances *C. elegans* survival against pathogen infection and increases pathogen clearance from the intestine.
- Fig. S2. NPR-8 functions in AVL and DVB neurons to regulate defecation but not survival against infection.
- Fig. S3. NPR-8 does not play a role in conserved innate immune pathways.
- Fig. S4. NPR-8–regulated collagen genes are involved in *C. elegans* defense and defecation.
- Fig. S5. NPR-8 regulates collagen expression in the cuticle and hypodermis and controls the dynamics of cuticle structure in response to infection.
- Fig. S6. NPR-8 is expressed in amphid sensory neurons and throughout developmental stages.
- Fig. S7. NPR-8 is not involved in bacteria sensing, as determined by food choice assays.
- Table S1. Differential expression of genes in conserved innate immune pathways in *npr-8(ok1439)* animals relative to wild-type animals exposed to *P. aeruginosa*.
- Table S2. Ontology analyses of up-regulated genes in *P. aeruginosa*–infected wild-type animals relative to uninfected controls or in uninfected *npr-8(ok1439)* animals relative to wild-type animals.
- Table S3. List of transgenic *C. elegans* strains generated in this study.

Download PDF

**Files in this Data Supplement:**

- Adobe PDF - aaw4717\_SM.pdf
