# Supplementary material for: Neuronal GPCR NPR-8 regulates C. elegans defense against pathogen infection
Source: Sci Adv. 2019 Nov 20;5(11):eaaw4717. doi: 10.1126/sciadv.aaw4717 (PMC6867885; doi:10.1126/sciadv.aaw4717)
Supplement: Download PDF [file aaw4717_SM.pdf]

Science Advances
AAAS

advances.sciencemag.org/cgi/content/full/5/11/eaaw4717/DC1

Supplementary Materials for

Neuronal GPCR NPR-8 regulates *C. elegans* defense against pathogen infection

Durai Sellegounder, Yiyong Liu, Phillip Wibisono, Chia-Hui Chen, David Leap, Jingru Sun*

*Corresponding author. Email: jingru.sun@wsu.edu

Published 20 November 2019, *Sci. Adv.* **5**, eaaw4717 (2019)
DOI: 10.1126/sciadv.aaw4717

This PDF file includes:

Fig. S1. Functional loss of NPR-8 enhances *C. elegans* survival against pathogen infection and increases pathogen clearance from the intestine.
Fig. S2. NPR-8 functions in AVL and DVB neurons to regulate defecation but not survival against infection.
Fig. S3. NPR-8 does not play a role in conserved innate immune pathways.
Fig. S4. NPR-8–regulated collagen genes are involved in *C. elegans* defense and defecation.
Fig. S5. NPR-8 regulates collagen expression in the cuticle and hypodermis and controls the dynamics of cuticle structure in response to infection.
Fig. S6. NPR-8 is expressed in amphid sensory neurons and throughout developmental stages.
Fig. S7. NPR-8 is not involved in bacteria sensing, as determined by food choice assays.
Table S1. Differential expression of genes in conserved innate immune pathways in *npr-8(ok1439)* animals relative to wild-type animals exposed to *P. aeruginosa*.
Table S2. Ontology analyses of up-regulated genes in *P. aeruginosa*–infected wild-type animals relative to uninfected controls or in uninfected *npr-8(ok1439)* animals relative to wild-type animals.
Table S3. List of transgenic *C. elegans* strains generated in this study.

SUPPLEMENTARY MATERIALS

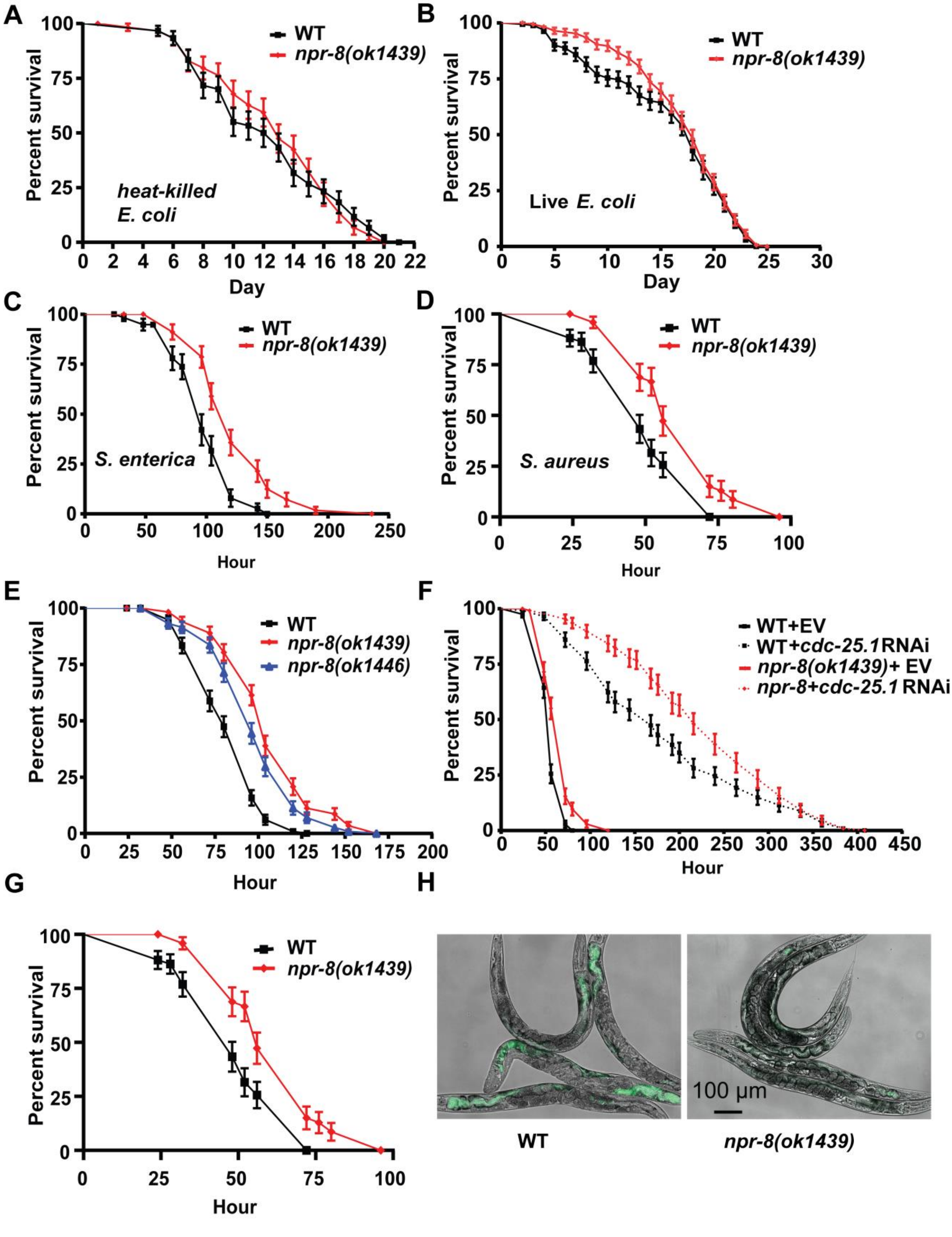

Fig. S1. Functional loss of NPR-8 enhances *C. elegans* survival against pathogen infection and increases pathogen clearance from the intestine. WT and *npr-8(ok1439)* animals were exposed to heat-killed *E. coli* strain OP50 (**A**), live *E. coli* OP50 (**B**), *Salmonella enterica* strain SL1344 (**C**), *Staphylococcus aureus* strain MSSA476 (**D**), and scored for survival over time. Assays for (A), (C) and (D) were performed at 25^{0}C to measure *C. elegans* survival against the respective bacteria; assays for (B) were performed at 20^{0}C to measure *C. elegans* lifespan on live *E. coli* OP50. The graphs are the combined results of three independent experiments. Each experiment included N = 60 adult animals per strain. Error bars represent the SEM. *P*-value represents the significance level of the mutant relative to the WT, $P = 0.962$ (A), $P < 0.0002$ (B), $P = 0.2707$ (C), and $P < 0.0001$ (D). (**E**), WT, *npr-8(ok1439),* and *npr-8(ok1446)* animals were exposed to *P. aeruginosa* and scored for survival over time. The graph is combined result of two independent experiments. Each experiment included N = 60 adult animals per strain. Error bars represent the SEM. *P*-value represents the significance level: WT vs *npr-8(ok1439), P* < 0.0001; WT vs *npr-8(ok1446), P* < 0.0001. (**F**), WT and *npr-8(ok1439)* animals grown on double-stranded RNA (dsRNA) for empty vector (EV) control or dsRNA for *cdc-25.1* were exposed to *P. aeruginosa* and scored for survival over time. The graphs are combined results of two independent experiments. Each experiment included N = 60 adult animals per strain. Error bars represent the SEM. *P*-value represents the significance level: WT+EV vs *npr-8(ok1439)*+EV, $P <$ 0.0001; WT+*cdc-25.1* RNAi vs *npr-8(ok1439)+cdc-25.1* RNAi, $P = 0.0118$. **(G),** WT and *npr-8(ok1439)* animals were exposed to *P. aeruginosa* in full lawn plates. The graphs are the combined results of three independent experiments. Each experiment included N = 60 adult animals per strain. Error bars represent the SEM. *P*-value of the mutant relative to the WT, $P =$ 0.0002. (**H**), WT and *npr-8(ok1439)* animals were exposed to GFP-expressing *P. aeruginosa* in full lawn plates for 24 hr and then visualized using a Zeiss Axio Imager M2 fluorescence microscope. Scale bar indicates 100 μm.

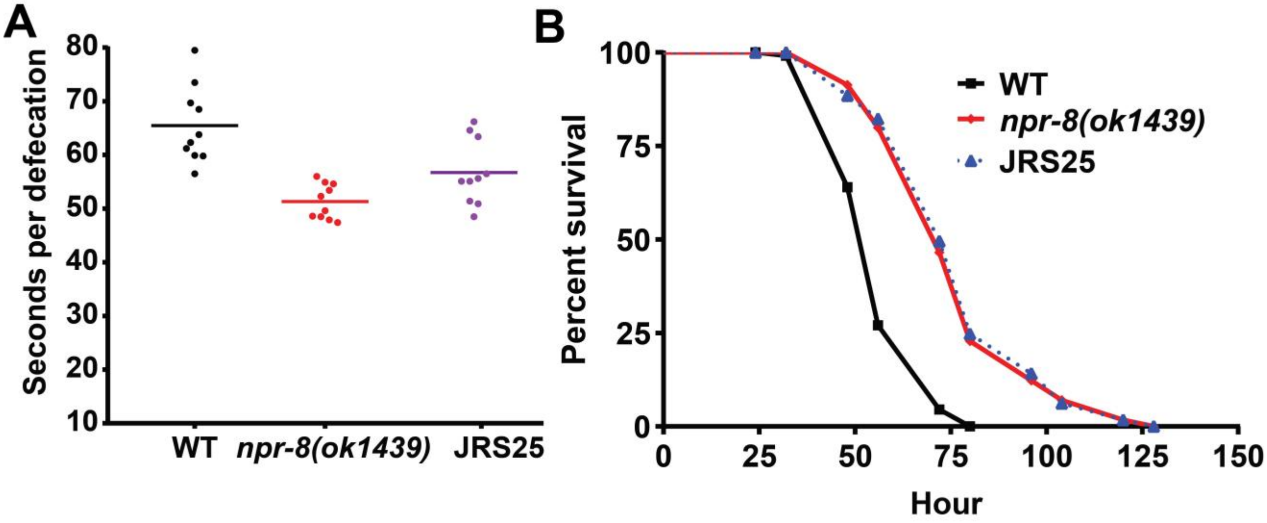

Fig. S2. NPR-8 functions in AVL and DVB neurons to regulate defecation but not survival against infection. (**A**), NPR-8 functions in AVL and DVB neurons to affect defecation. WT, *npr-8(ok1439)* and JRS25 animals were exposed to *P. aeruginosa* in full lawn plates for 24 hours. Defecation rates of animals were measured as the averages of 10 intervals between two defecation cycles. *N* = 10 animals per strain were used for the measurements. JRS25, a strain with *npr-8* expression recued in AVL and DVB neurons in *npr-8(ok1439)* animals. *P*-value represents the significance level: WT vs *npr-8*, $P < 0.001$; WT vs JRS25, $P = 0.008$; *npr-8* vs JRS25, $P = 0.024$. (**B**), NPR-8 does not function in AVL and DVB neurons to regulate survival against infection. WT, *npr-8(ok1439)* and JRS25 animals were exposed to *P. aeruginosa* and scored for survival over time. The graph is a combined result of two independent experiments. Each experiment included *N* = 60 adult animals per strain. *P*-value represents the significance level: WT vs *npr-8*, $P < 0.0001$; WT vs JRS25, $P < 0.0001$; *npr-8* vs JRS25, $P = 0.7382$.

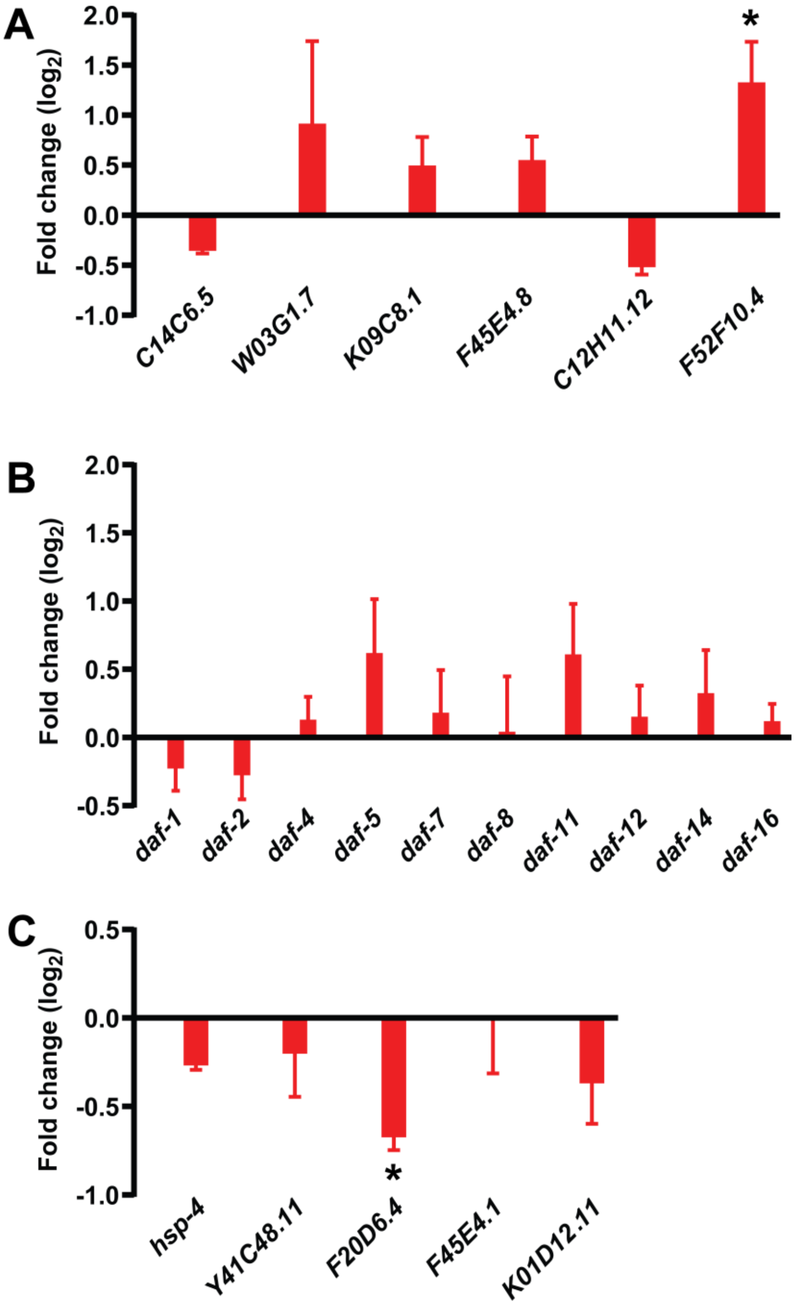

Fig. S3. NPR-8 does not play a role in conserved innate immune pathways. qRT-PCR was performed to measure gene expression in the PMK-1 pathway (**A**), the TGF-β pathway (**B**), and the UPR pathway **(C)** in WT and *npr-8(ok1439)* animals exposed to *P. aeruginosa* for 4 hr. The graphs are the combined results of three independent experiments. Error bars represent SEM. Asterisks (*) denote a significant difference (*P* < 0.05) between the WT and mutant animals.

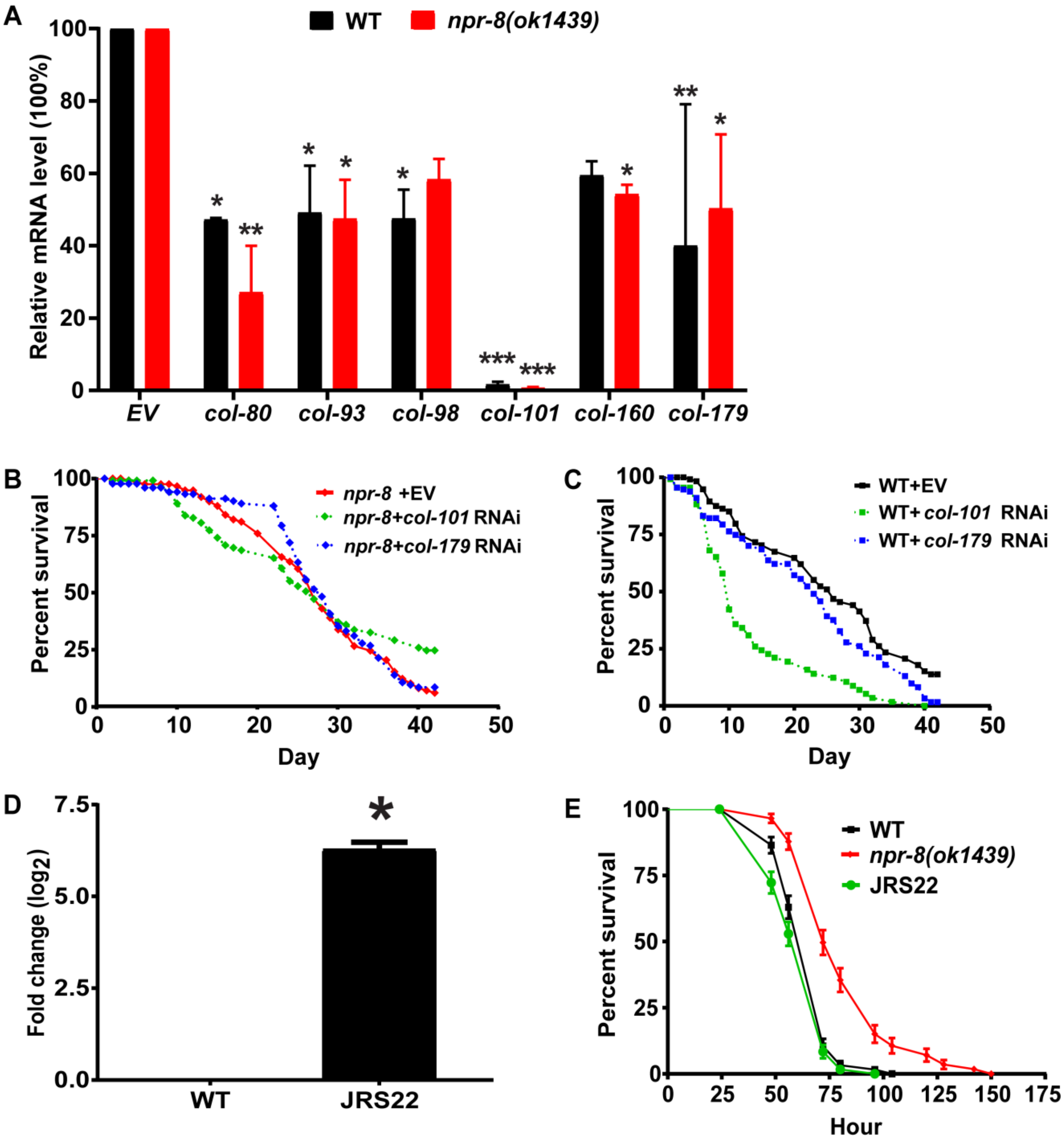
A
WT
npr-8(ok1439)
Relative mRNA level (100%)
100
80
60
40
20
0
EV
col-80
col-93
col-98
col-101
col-160
col-179
B
npr-8 +EV
npr-8+col-101 RNAi
npr-8+col-179 RNAi
Percent survival
Day
C
WT+EV
WT+col-101 RNAi
WT+col-179 RNAi
Percent survival
Day
D
Fold change (log2)
7.5
5.0
2.5
0.0
WT
JRS22
E
WT
npr-8(ok1439)
JRS22
Percent survival
Hour

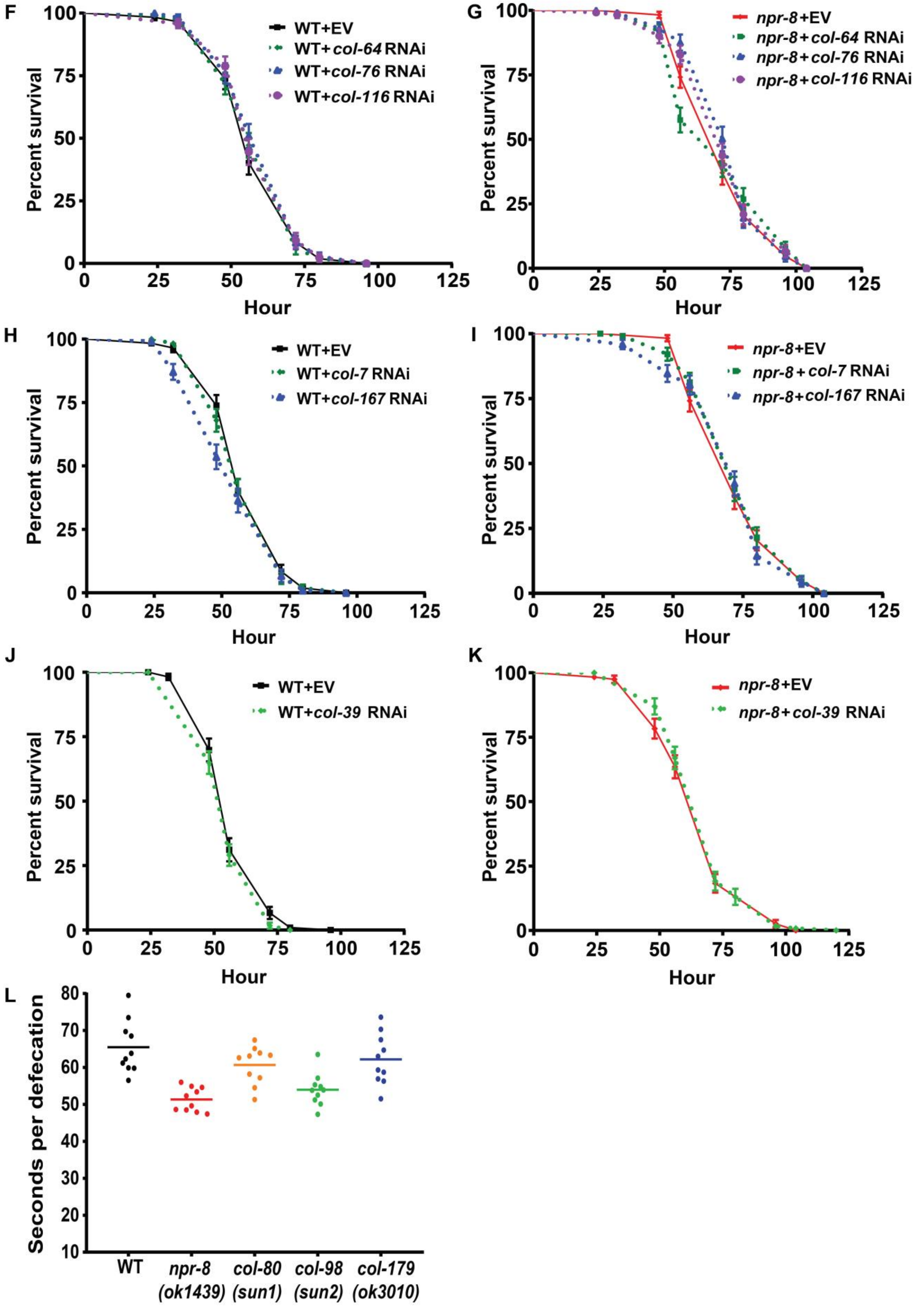
F
WT+EV
WT+col-64 RNAi
WT+col-76 RNAi
WT+col-116 RNAi
Percent survival
Hour
G
npr-8+EV
npr-8+col-64 RNAi
npr-8+col-76 RNAi
npr-8+col-116 RNAi
H
WT+EV
WT+col-7 RNAi
WT+col-167 RNAi
I
npr-8+EV
npr-8+col-7 RNAi
npr-8+col-167 RNAi
J
WT+EV
WT+col-39 RNAi
K
npr-8+EV
npr-8+col-39 RNAi
L
Seconds per defecation
WT
npr-8 (ok1439)
col-80 (sun1)
col-98 (sun2)
col-179 (ok3010)

Fig. S4. NPR-8–regulated collagen genes are involved in *C. elegans* defense and defecation. (**A**), Expression of collagen genes was efficiently knocked down by RNAi. qRT-PCR analysis was performed to measure the expression of collagen genes in WT and *npr-8(ok1439)* animals. Bars represent mean ± SEM, and values are the average of two independent experiments. Expression of the collagen genes was analyzed after exposure to corresponding double stranded RNAi clones. Expression levels were calculated relative to empty vector (EV). In all assays, pan-actin served as internal controls. Asterisks (*) denote a significant difference ($P < 0.05$) between the EV and the gene-specific RNAi. (**B**, **C**), Collagens affect *C. elegans* lifespan on heat-killed *E. coli* OP50. *npr-8(ok1439)* and WT animals grown on dsRNA for empty vector (EV) control or dsRNA for a collagen gene (*col-101* or *col-179)* were exposed to heat-killed *E. coli* OP50 and scored for survival over time. The graphs are combined results of two independent experiments. Each experiment included $N = 60$ adult animals per strain. *P*-value represents the significance level. *P* values in (B) are relative to *npr-8*+EV*: npr-8+col-101* RNAi, $P = 0.1010$; *npr-8+col-179* RNAi, $P = 0.5465$. *P* values in (C) are relative to WT+ EV: WT+*col-101* RNAi, $P < 0.0001$; WT+*col-179* RNAi, $P = 0.0168$. (**D**), *col-179* was over-expressed in transgenic strain JRS22. qRT-PCR analysis was performed to measure *col-179* expression in WT and JRS22 animals. The graphs are combined results of two independent experiments. Bars represent mean ± SEM. Asterisks (*) denote a significant difference ($P < 0.0001$) in expression between WT and JRS22 animals. (**E**), Collagen overexpression did not mimic the enhanced survival phenotype. WT, *npr-8(ok1439)* and JRS22 animals were exposed to *P. aeruginosa* and scored for survival over time. The graphs are combined results of two independent experiments. Each experiment included $N = 60$ adult animals per strain. Error bars represent the SEM. *P*-value represents the significance level: WT vs *npr-8(ok1439)*, $P < 0.0001$; WT vs JRS22, $P = 0.05$; *npr-8(ok1439)* vs JRS22, $P < 0.0001$. (**F**-**K**), Unrelated collagen genes or collagen genes enriched in non-pathogenic conditions are not required for *C. elegans* defense against *P. aeruginosa* infection. WT and *npr-8(ok1439)*

animals grown on ds RNA for empty vector (EV) control or dsRNA for collagen genes were exposed to *P. aeruginosa* and scored for survival over time. The graphs are combined results of two independent experiments. Each experiment included *N* = 60 adult animals per strain. Error bars represent the SEM. *P*-value represents the significance level. *P* values in (F) are relative to WT+EV: WT+*col-64* RNAi, *P* = 0.6975; WT+*col-76* RNAi, *P* = 0.2323; WT+*col-116* RNAi, *P* = 0.4688. *P* values in (G) are relative to *npr-8*+EV*: npr-8+col-64* RNAi, *P* = 0. 9586; *npr-8+col-76* RNAi, *P* = 0.2223; *npr-8+col-116* RNAi, *P* =0.3978. *P* values in (H) are relative to WT+EV: WT+*col-7* RNAi, *P* = 0.6835; WT+*col-167* RNAi, *P* = 0.0819. *P* values in (I) are relative to *npr-8*+EV*: npr-8+col-7* RNAi, *P* = 0.6280; *npr-8+col-167* RNAi, *P* = 0.8806. *P* value in (J) is relative to WT+EV: WT+*col-39* RNAi, *P* = 0.2966. *P* value in (K) is relative to *npr-8*+EV*: npr-8+col-39* RNAi, *P* = 0.3194. (**L**), Mutations in collagen genes affect defecation. WT, *npr-8(ok1439), col-80(sun-1), col-98(sun-2)* and *col-179(ok3010)* animals were exposed to *P. aeruginosa* in full lawn plates for 24 hrs. Defecation rates of the animals were measured as the averages of 10 intervals between defecation cycles. *N* = 10 animals per strain were used for the measurements. Error bars represent the SEM. *P*-value represents the significance level: WT vs *npr-8(ok1439), P* < 0.001; WT vs *col-80(sun1), P* = 0.1015; *npr-8(ok1439)* vs *col-80(sun1), P* = 0.0001; WT vs *col-98(sun2), P* = 0.0004; *npr-8(ok1439)* vs *col-98(sun2), P* = 0.1452; WT vs *col-179(ok3010), P* > 0.05; *npr-8(ok1439)* vs *col-179(ok3010), P* < 0.01.

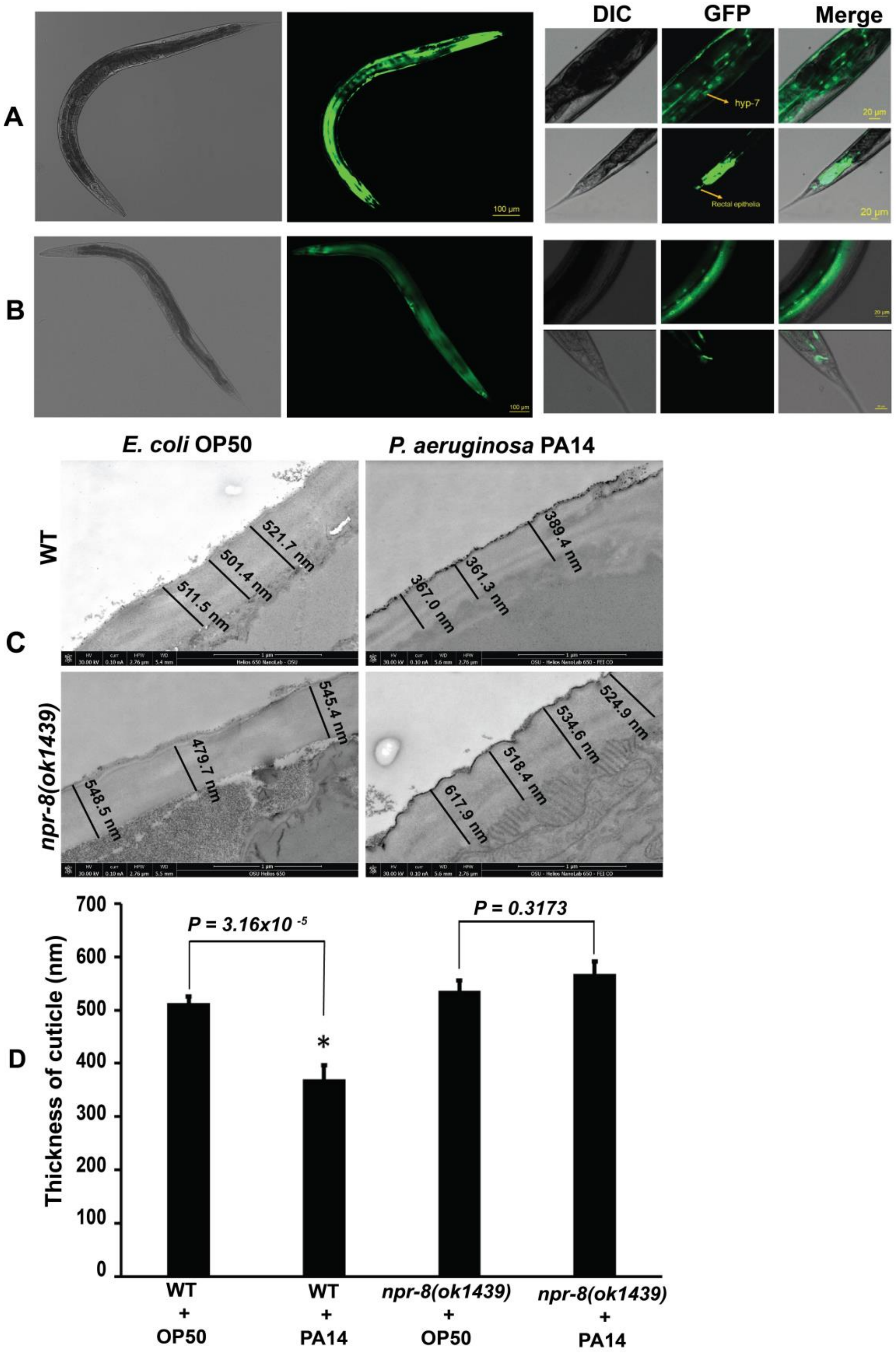

A
B
C
D
DIC
GFP
Merge
hyp-7
Rectal epithelia
20 µm
100 µm
E. coli OP50
P. aeruginosa PA14
WT
npr-8(ok1439)
521.7 nm
501.4 nm
511.5 nm
389.4 nm
361.3 nm
367.0 nm
545.4 nm
479.7 nm
548.5 nm
524.9 nm
534.6 nm
518.4 nm
617.9 nm
Thickness of cuticle (nm)
P = 3.16x10 -5
P = 0.3173
*
WT + OP50
WT + PA14
npr-8(ok1439) + OP50
npr-8(ok1439) + PA14

Fig. S5. NPR-8 regulates collagen expression in the cuticle and hypodermis and controls the dynamics of cuticle structure in response to infection. (**A**, **B**), NPR-8-regulated collagens are expressed in the cuticle and hypodermis. Fluorescent images of *C. elegans* reporter strains expressing translational reporter (*col-179p::col-179gDNA::gfp*) (A) or transcriptional reporter (*col-101p::SL2::gfp*) (B) show collagen genes *col-179* and *col-101* are expressed in the cuticle, hypodermal cells (hyp-7) and rectal epithelial cells. (**C**, **D**), NPR-8 controls the dynamics of cuticle structure in response to infection. WT and *npr-8(ok1439)* animals were exposed to *E. coli* OP50 or *P. aeruginosa* PA14 for 24 hours, and the ultrastructure of cuticle structure was analyzed using Transmission Electron Microscopy (TEM). For each experimental group (WT+OP50, WT+PA14, *npr-8*+OP50, or *npr*-8+PA14), 20-25 cross-sections from the midbody region were observed, and representative images were presented (C). The thickness of the cuticle was measured across the entire cuticular layers and compared between experimental groups (D). Three to four measurements were done for each cross-section and averaged to give a thickness value. Each bar represents the mean of thickness values from 20-25 cross-sections. Error bars represent SEM. *P*-value represents the significance level of the indicated comparison. Asterisk (*) denotes a significant difference ($P < 0.05$).

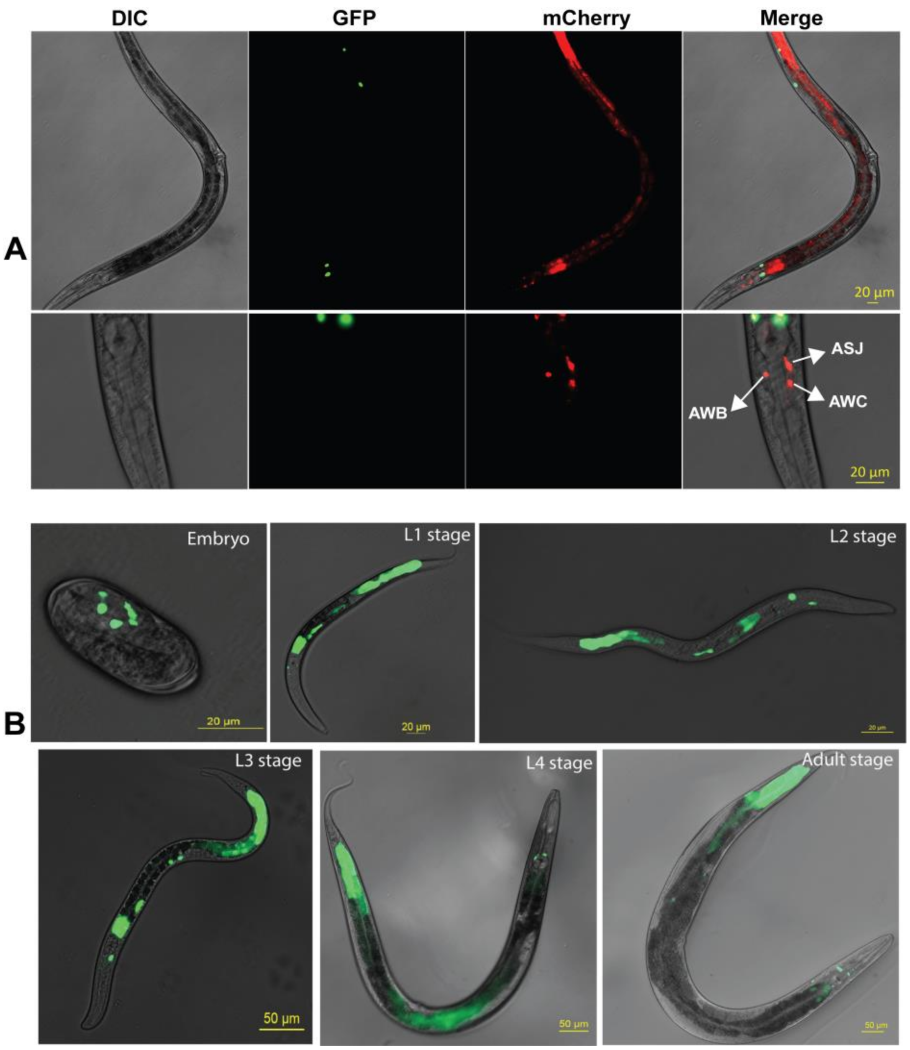

Fig. S6. NPR-8 is expressed in amphid sensory neurons and throughout developmental stages. (**A**), NPR-8 is expressed in the amphid sensory neurons AWB, ASJ and AWC and the intestine. Transcriptional reporter strain *npr-8p::mCherry* expresses mCherry under the control of the *npr-8* promoter and expresses GFP from co-injection marker *unc-122p::gfp*. DIC, differential interference contrast microscopy. GFP, GFP fluorescence microscopy. mCherry, mCherry fluorescence microscopy. Merge, overlay of DIC, GFP and mCherry images. (**B**), Expression of *npr-8p::gfp* reporter at different developmental stages. Fluorescent images of *npr-8p::gfp* reporter at embryo, L1 larva, L2 larva, L3 larva, L4 larva and young adult stages. In all developmental stages except embryo, expression of GFP was observed in three pairs of head neurons (AWB, AWC and ASJ) and the lower gut region.

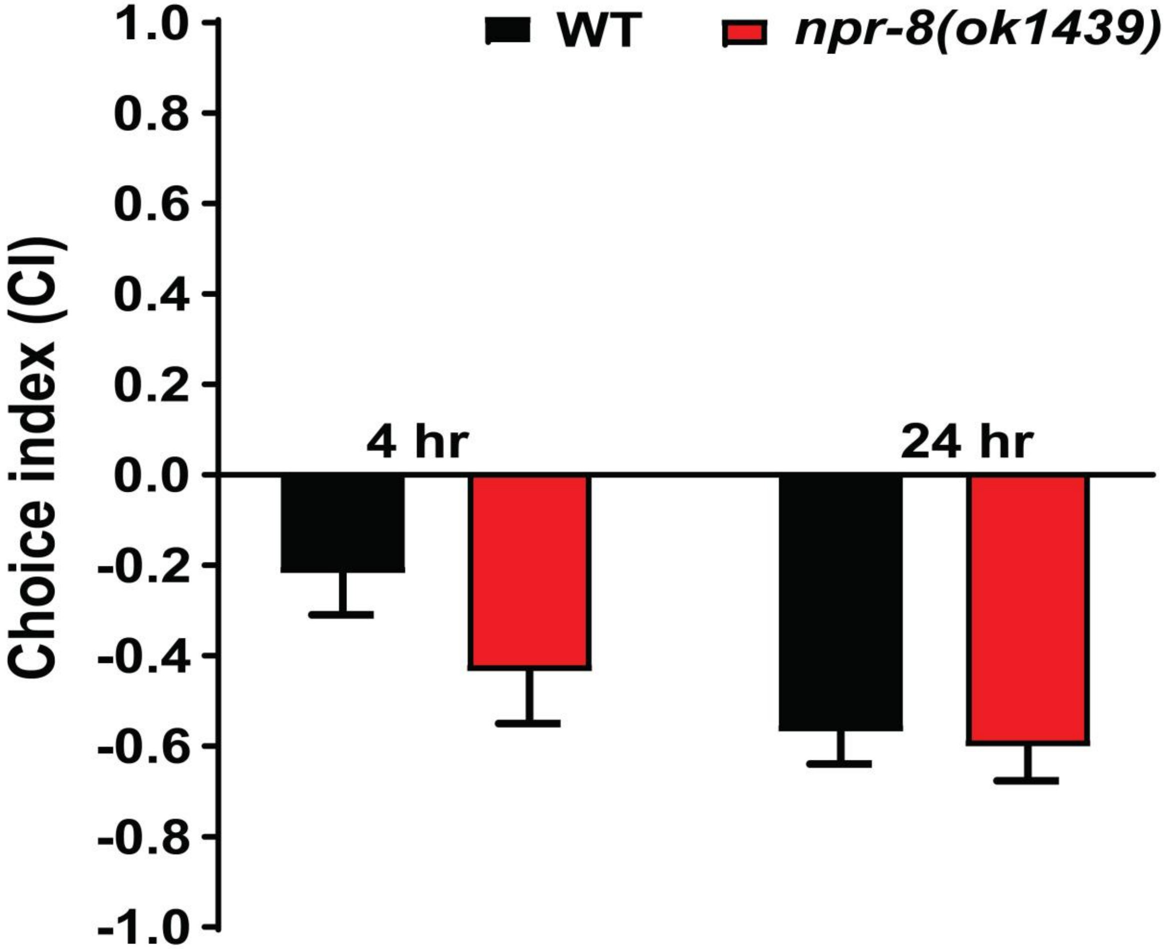

Fig. S7. NPR-8 is not involved in bacteria sensing, as determined by food choice assays. WT and *npr-8(ok1439)* animals were placed on plates containing spots of two types of bacteria: *E. coli* OP50 and *P. aeruginosa* PA14. At 4 hr and 24 hr, the distributions of the animals on these bacteria were counted and calculated to give a Choice Index (CI = (number of animals on *P. aeruginosa* – number of animals on *E. coli*) / total number of animals). The graph represents the combined results of three independent experiments. Error bars represent SEM. *P*-values represent the significance level of the mutants relative to the WT, 4 hr, $P = 0.219766$; 24 hr, $P = 0.767644$.

Table S1. Differential expression of genes in conserved innate immune pathways in *npr-8(ok1439)* animals relative to wild-type animals exposed to *P. aeruginosa*.

Top 10 most highly regulated genes in each of the three conserved innate immune pathways reported by previous studies		***npr-8(ok1439)*** **animals relative to wild-type animals***
Gene Name	Fold Change	Fold Change
**the PMK-1 pathway (from Table 1 in Troemel *et al.* 2006 PLoS Genet 2, e183)**		
F08G5.6	29	1.7
C09H5.2 / catp-3	25	1.7
C17H12.8	17	1.1
F01D5.5	11	1.6
Y22F5A.5 / lys-2	8	0.8
F01D5.1	8	1.8
F55G11.8	7	1.1
T24B8.5	7	1.7
F59A7.2	6	1.2
K04F1.9	6	1.3
**the TGF-β pathway (from Table 2 in Mochii *et al.* 1999 PNAS 96, 15020-15025)**		
T01A4.1 / gcy-28	3.48	0.9
D1014.5	2.08	1.5
C08E3.13	0.02	1.2
F42A8.1	0.17	1.3
K01A2.3	0.25	1.0
F09C8.1	0.26	0.7
F35C5.8 / clec-65	0.33	0.9
F46F2.3	0.35	1.3
F35C5.6 / clec-63	0.37	0.7
ZK1320.3	0.37	0.9
**the UPR pathway (from table S1 in Shen *et al.* 2005 PLoS Genet 1, e37)**		
Y41C4A.11	4.94	1.4
ARL-1	4.15	0.9
HSP-4	3.72	1.0
C47B2.6 / gale-1	3.58	0.9
B0285.9 / ckb-2	3.55	1.0
C37A5.8 / fipr-24	3.43	1.4
ARL-7 / F20D1.5	3.37	NA
F07A11.2 / gfat-1	3.37	1.1
C04F12.1	3.26	1.1
T28F3.3 / zipt-7.1	3.22	NA

* Data of gene differential expression were extracted from our RNA-sequencing data (GEO accession number GSE122544, processed data file, N2PA14_vs_npr8PA14.deseq.results.csv)

Table S2. Ontology analyses of up-regulated genes in *P. aeruginosa*–infected wild-type animals relative to uninfected controls or in uninfected *npr-8(ok1439)* animals relative to wild-type animals.

(**A**), Enrichment of biological processes revealed by gene ontology analysis of *P. aeruginosa*-induced genes in wild-type animals

GO term	Description	P-value[#]	FDR q-value*	Enrichment (N, B, n, b)[§]
**GO:0002376**	immune system process	3.72E-42	1.01E-38	3.76 (2646,191,398,108)
**GO:0006955**	immune response	1.77E-41	2.41E-38	3.74 (2646,190,398,107)
**GO:0045087**	innate immune response	1.77E-41	1.61E-38	3.74 (2646,190,398,107)
**GO:0006952**	defense response	9.85E-39	6.72E-36	3.24 (2646,248,398,121)
**GO:0006950**	response to stress	6.17E-34	3.37E-31	2.70 (2646,336,405,139)
**GO:0050896**	response to stimulus	3.04E-31	1.38E-28	2.43 (2646,412,405,153)
**GO:0042742**	defense response to bacterium	9.93E-08	3.87E-05	2.60 (2646,71,502,35)
**GO:0009617**	response to bacterium	9.93E-08	3.38E-05	2.60 (2646,71,502,35)
**GO:0050829**	defense response to Gram-negative bacterium	4.76E-07	1.44E-04	2.73 (2646,56,502,29)
**GO:0043207**	response to external biotic stimulus	5.59E-07	1.52E-04	2.43 (2646,78,502,36)
**GO:0098542**	defense response to other organism	5.59E-07	1.38E-04	2.43 (2646,78,502,36)
**GO:0009607**	response to biotic stimulus	5.59E-07	1.27E-04	2.43 (2646,78,502,36)
**GO:0051707**	response to other organism	5.59E-07	1.17E-04	2.43 (2646,78,502,36)
**GO:0050907**	detection of chemical stimulus involved in sensory perception	9.96E-06	1.94E-03	11.13 (2646,16,104,7)
**GO:0051704**	multi-organism process	2.84E-05	5.17E-03	2.13 (2646,89,502,36)
**GO:0009593**	detection of chemical stimulus	9.42E-05	1.61E-02	8.48 (2646,21,104,7)
**GO:0097501**	stress response to metal ion	2.89E-04	4.64E-02	3.03 (2646,9,874,9)
**GO:0030968**	endoplasmic reticulum unfolded protein response	3.43E-04	5.20E-02	3.37 (2646,16,540,11)
**GO:0050906**	detection of stimulus involved in sensory perception	4.36E-04	6.25E-02	6.85 (2646,26,104,7)
**GO:0009605**	response to external stimulus	4.89E-04	6.67E-02	3.31 (2646,121,99,15)
**GO:1990169**	stress response to copper ion	8.39E-04	1.09E-01	17.41 (2646,4,114,3)

[#] P-value is computed according to the mHG model (Eden *et al.* 2007 PLoS Comp Bio 3(3):e39).

* FDR q-value is the correction of the above p-value for multiple testing using the Benjamini and Hochberg method (Benjamini and Hochberg 1995 J R Statist Soc B 57(1):289-300).

[§] Enrichment (N, B, n, b) is defined as follows:
N - total number of genes
B - total number of genes associated with a specific GO term
n - number of genes in the target set
b - number of genes in the intersection
Enrichment = (b/n) / (B/N)

(**B**), Enrichment of molecular functions revealed by gene ontology analysis of upregulated genes in uninfected *npr-8(ok1439)* animals relative to wild-type animals

**GO term**	**Description**	**P-value[#]**	**FDR q-value***	**Enrichment (N, B, n, b)[§]**
GO:0042302	structural constituent of cuticle	1.92E-46	4.28E-43	34.27 (10272,122,86,35)
GO:0005198	structural molecule activity	2.78E-29	3.1E-26	11.91 (10272,351,86,35)

(**C**), Enrichment of cuticle structure genes in uninfected *npr-8(ok1439)* animals relative to wild-type controls

**Enriched cuticle structure genes**	**Fold change**	**Adjusted *p* value**#
col-147	5.2	2.31E-47
col-167	5.2	1.85E-46
col-7	4.9	6.45E-41
col-39	4.5	1.17E-53
col-107	4.5	2.94E-39
col-62	4.2	6.87E-37
col-74	3.8	1.13E-27
col-165	3.5	3.13E-24
col-149	3.4	1.20E-23
col-146	3.3	9.24E-23
col-168	3.2	7.43E-21
col-125	3.1	1.06E-21
col-133	3.0	2.91E-18
col-65	2.9	3.38E-17
cut-2	2.9	8.31E-18
col-77	2.8	1.59E-16
col-180	2.7	8.74E-16
col-81	2.6	6.41E-14
col-109	2.6	6.51E-15
col-130	2.6	1.10E-14
col-13	2.5	4.68E-14
col-139	2.4	2.90E-12
col-14	2.4	4.08E-13
dpy-4	2.3	3.12E-11
col-12	2.2	4.45E-10
col-161	2.2	6.94E-11
col-169	2.2	3.13E-10
col-49	2.1	5.00E-09
dpy-13	2.1	5.60E-09
col-97	2.1	1.62E-09
col-129	2.1	4.41E-09
col-138	2.1	1.55E-09
col-104	2.0	5.11E-09
bli-6	2.0	6.99E-08
dpy-5	2.0	3.54E-08

Adjusted *p* value is the correction of the *p* value for multiple testing using the Benjamini and Hochberg method (Benjamini and Hochberg 1995 J R Statist Soc B 57(1):289-300).

Table S3. List of transgenic *C. elegans* strains generated in this study.

Strain	Plasmid and genetic background	Host	Note
JRS13	pSD01 *npr-8p(2kb)::gfp*	N2	*npr-8* transcriptional reporter
JRS14	pSD02 *npr-8p(2kb)::mCherry*	N2	*npr-8* transcriptional reporter
JRS17	pSDG04 *npr-8p(2kb)::npr-8 gDNA::gfp*	*npr-8(ok1439)*	*npr-8* genomic rescue driven by *npr-8* promoter
JRS18	pSDG05 *str-1p(1.3kb)::npr-8 gDNA::gfp*	*npr-8(ok1439)*	*npr-8* genomic rescue in AWB neurons driven by *str-1* promoter
JRS19	pSDG06 *str-2p(2kb)::npr-8 gDNA::gfp*	*npr-8(ok1439)*	*npr-8* genomic rescue in AWC neurons driven by *str-2* promoter
JRS20	pSDG07 *trx-1p(1.1kb)::npr-8 gDNA::gfp*	*npr-8(ok1439)*	*npr-8* genomic rescue in ASJ neurons driven by *trx-1* promoter
JRS21	pSDG08 *rgef-1p(3.4kb)::npr-8 gDNA::gfp*	*npr-8(ok1439)*	pan neuronal *npr-8* genomic rescue driven by *rgef-1* promoter
JRS22	pSDG09 *col-179p(965bp)::col-179 gDNA::gfp*	N2	*col-179* gene over-expression in N2 animals
JRS24	pSDG10 *ges-1p(2.1kb)::npr-8 gDNA::gfp*	*npr-8(ok1439)*	*npr-8* genomic rescue in intestine driven by *ges-1* promoter
JRS25	pSDG11 *aex-2p(2kb)::npr-8 gDNA::gfp*	*npr-8(ok1439)*	*npr-8* genomic rescue in GABA neurons driven by *aex-2* promoter
JRS26	pSD03 *col-101p(2.1kb)::gfp*	N2	*col-101* transcriptional reporter
JRS30	*npr-8(ok1439);col-179(ok3010)*		Double mutant for *npr-8(ok1439)* and *col-179(ok3010)*
JRS37	*col-80(sun1)*	N2	CRISPR edited mutant strain for *col-80* (deleted region 1171bp)
JRS38	*col-98(sun2)*	N2	CRISPR edited mutant strain for *col-98* (deleted region 1203bp)
JRS39	*npr-8(ok1439);col-80(sun1)*		Double mutant for *npr-8(ok1439)* and *col-80(sun1)*
JRS40	*npr-8(ok1439);col-98(sun2)*		Double mutant for *npr-8(ok1439)* and *col-98(sun2)*

* Please refer to section "Plasmid construction and transgenic animal generation" in **Materials and Methods** for detailed information about transgenic animal generation.
